# Estimating PM2.5-related premature mortality and morbidity associated with future wildfire emissions in the western US

**DOI:** 10.1088/1748-9326/abe82b

**Published:** 2021-03-08

**Authors:** James E Neumann, Meredith Amend, Susan Anenberg, Patrick L Kinney, Marcus Sarofim, Jeremy Martinich, Julia Lukens, Jun-Wei Xu, Henry Roman

**Affiliations:** 1 Industrial Economics, Inc., Cambridge, MA, United States of America; 2 George Washington University, Washington, DC, United States of America; 3 School of Public Health, Boston University, Boston, MA, United States of America; 4 US Environmental Protection Agency, Washington, DC, United States of America; 5 Dalhousie University, Halifax, Nova Scotia, Canada

**Keywords:** wildfire, climate change, premature mortality, morbidity, economic valuation

## Abstract

Wildfire activity in the western United States (US) has been increasing, a trend that has been correlated with changing patterns of temperature and precipitation associated with climate change. Health effects associated with exposure to wildfire smoke and fine particulate matter (PM_2.5_) include short- and long-term premature mortality, hospital admissions, emergency department visits, and other respiratory and cardiovascular incidents. We estimate PM_2.5_ exposure and health impacts for the entire continental US from current and future western US wildfire activity projected for a range of future climate scenarios through the 21st century. We use a simulation approach to estimate wildfire activity, area burned, fine particulate emissions, air quality concentrations, health effects, and economic valuation of health effects, using established and novel methodologies. We find that climatic factors increase wildfire pollutant emissions by an average of 0.40% per year over the 2006–2100 period under Representative Concentration Pathway (RCP) 4.5 (lower emissions scenarios) and 0.71% per year for RCP8.5. As a consequence, spatially weighted wildfire PM_2.5_ concentrations more than double for some climate model projections by the end of the 21st century. PM_2.5_ exposure changes, combined with population projections, result in a wildfire PM2.5-related premature mortality excess burden in the 2090 RCP8.5 scenario that is roughly 3.5 times larger than in the baseline period. The combined effect of increased wildfire activity, population growth, and increase in the valuation of avoided risk of premature mortality over time results in a large increase in total economic impact of wildfire-related PM_2.5_ mortality and morbidity in the continental US, from roughly $7 billion per year in the baseline period to roughly $36 billion per year in 2090 for RCP4.5, and $43 billion per year in RCP8.5. The climate effect alone accounts for a roughly 60% increase in wildfire PM2.5-related premature mortality in the RCP8.5 scenario, relative to baseline conditions.

## 1. Introduction

The annual number and size of wildfires in the western United States (US) have been increasing [1–3]. Recent trends are a significant departure from historic wildfire regimes and are significantly correlated with human-induced climate change [4–8]. Wildfire smoke degrades air quality both near fires and far downwind—a phenomenon readily apparent in the 2020 wildfire season [9, 10]. The intensity of the 2018 wildfires in the western US led to five rural cities in northern California and the Pacific Northwest registering annual average fine particulate matter (PM_2.5_) concentrations exceeding 20 *μ*g m^*−*3^, compared with the National Ambient Air Quality Standard of 12 *μ*g m^*−*3^ [11].

Climate change affects wildfire activity by modifying patterns of temperature and precipitation, which can increase the number and length of low moisture periods in air, soil, and biomass [1, 3, 5, 7, 12–14]. Climate effects vary geographically depending on whether an area is fuel-limited or flammability-limited [12]. Most studies examining these patterns in the western US have found that the largest increases in wildfire activity are likely to occur in northern California and the intermountain West—areas with high fuel loads that are projected to experience increased aridity [6]. In addition, lightning strikes in the contiguous US (CONUS) are projected to increase at higher temperatures—up to 50% over this century [15]. Other non-climate drivers of wildfire activity in the western US include the role of changing settlement patterns in the wildland–urban interface and human wildfire ignitions [16, 17].

As wildfire activity increases, the effects of wildfire emissions on local and downwind communities will also intensify [9, 18–20]. Exposure to wildfire smoke can lead to significant health effects that impose a substantial cost on society [21–23]. Previous studies have linked exposure to wildfire smoke with a broad range of health effects, including premature mortality, hospital admissions, and emergency department (ED) visits, often focused on respiratory ailments [9, 10, 24–26]. The literature on the potential air pollution-related mortality and morbidity burden from future wildfire smoke exposure in the US remains limited, though appears relatively consistent in estimating worsening health outcomes with some heterogeneity by region, scenario, and climate model used [10, 24].

Here we build on this earlier work to provide a comprehensive analysis of the present and potential future health burden associated with PM_2.5_ exposure from wildfire smoke in the western US. Specifically, we examine the health burden of projected future wildfire activity on P_2.5_-attributable premature mortality and a range of morbidity outcomes, including hospital admissions, ED visits, asthma exacerbations, and upper and lower respiratory symptoms (see table 1 and, for details on specific endpoints and ICD codes, table S7 in the supplemental material (available online at stacks.iop.org/ERL/16/035019/mmedia)). We use methods compatible with the US Environmental Protection Agency’s (US EPA) Climate Change Impacts and Risk Analysis (CIRA) framework, developed as an input to the US Global Change Research Program’s National Climate Assessment, to achieve two distinct objectives: (a) to allow comparison of the results with the economic impacts of climate change on other sectors in the US; and (b) to assess wildfire impacts across two future climate forcing scenarios simulated in five climate models [27, 28]. Assessing a range of outcomes across greenhouse gas emissions (GHGs) scenarios and alternative climate models adds new insights relative to previous wildfire literature, which have typically relied on a single general circulation model (GCM—a model with a global domain that projects future multidimensional climate change) or single GHG emission scenario. Our results also complement other recent CIRA estimates that use similar methods to show climate change could also alter meteorological conditions in ways that still further increase US population exposure to PM_2.5_ beyond that estimated here [29].

## 2. Methods

**Figure 1.**
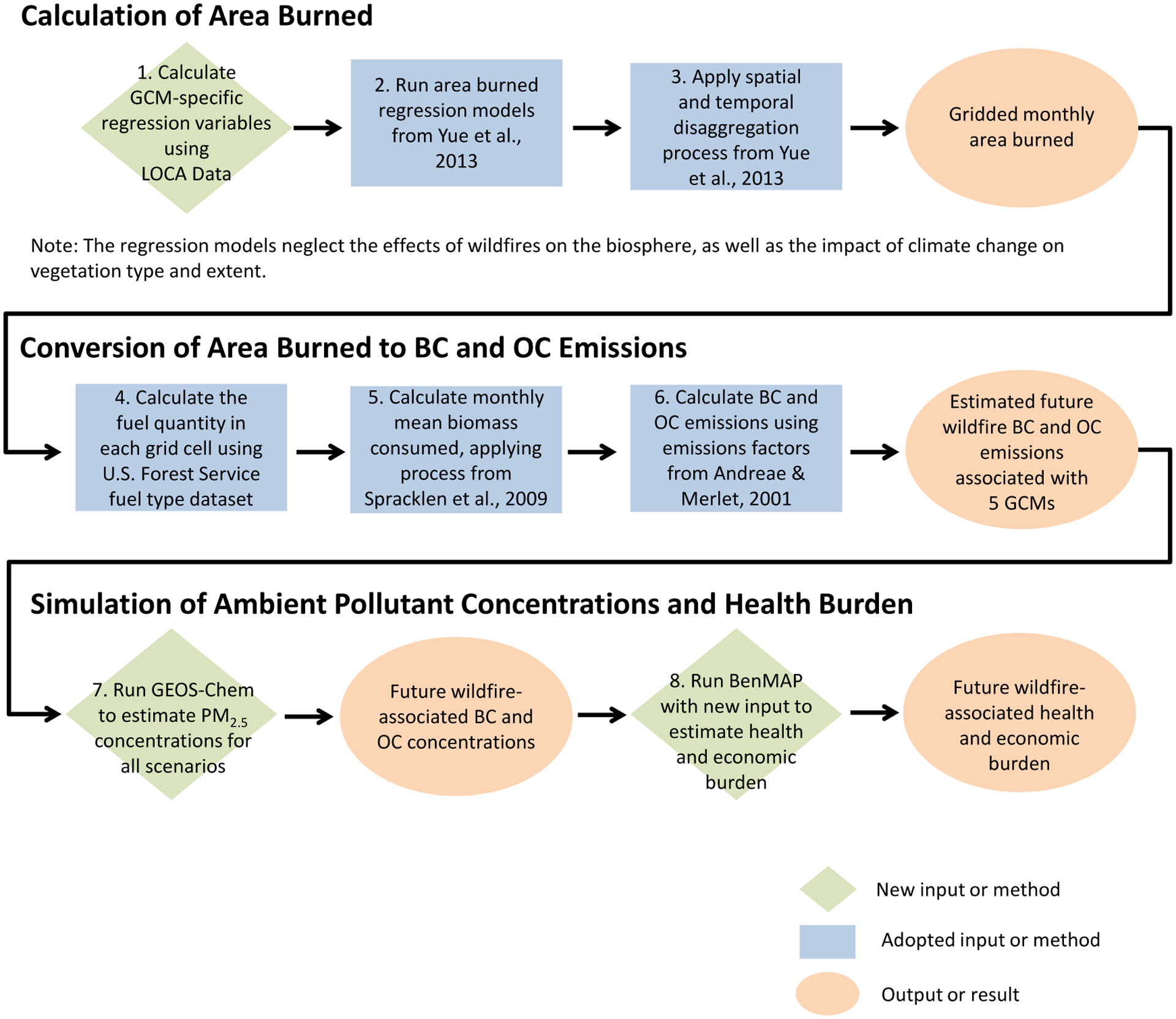
Key analytical steps, from GCM-based climate inputs at step 1 in upper left through calculation of area burned, conversion of area burned to BC and OC air pollutant emissions, simulation of ambient pollutant concentration with GEOS-Chem model, and estimation of health and economic burden using the BenMAP air pollution health effects and valuation model to estimate mortality and morbidity incidence and economic valuation outputs.

We estimate wildfire-attributable PM_2.5_ and associated mortality and morbidity in the US under two climate scenarios and five GCMs throughout the 21st century. The two climate scenarios are Representative Concentration Pathway (RCP) 8.5, a higher GHGs scenario, and RCP4.5, a lower scenario. We do not assign probabilities to the relative likelihood that one of these scenarios may describe better the pathway of future emissions. We employ spatially downscaled climate projections from the Localized Constructed Analogs dataset for five Earth system models participating in the Coupled Model Intercomparison Project Phase 5: CanESM2, CCSM4, GISS-E2-R, HadGEM2-ES, and MIROC5, consistent with the CIRA framework (described further in section 1 of supplemental material) [27, 28]. We average across 10 year climatic time periods to smooth interannual variability: a baseline of 1996–2005, and projections for 2050 (2046–2055) and 2090 (2086–2095). For each climate scenario, GCM, and year, we: (a) estimate ecoregion-specific wildfire area burned and black carbon (BC) and organic carbon (OC) emissions (see supplemental material sections 2 and 3); (b) simulate ambient PM_2.5_ concentrations resulting from these emissions; and (c) quantify wildfire-attributable PM_2.5_ mortality and morbidity (figure 1). We estimate wildfire activity in the western US only, because the high prevalence of prescribed burns in the east obscures statistical relationships between climate and wildfires. However, we estimate air quality and health impacts stemming from western wildfire-related PM_2.5_ for the entire CONUS in order to provide a more comprehensive estimate of the domestic effects of wildfire changes, since smoke can travel long distances, affecting air quality on continental scales.

### 2.1. Estimating annual area burned and wildfire emissions based on climate data

We estimate fire activity and associated emissions using previously published regression models for the western US that relate historical annual area burned with meteorological variables and fire indexes [5]. Yue *et al* provides a set of equations that relate climatic conditions to wildfire area burned for six ecoregions covering the contiguous western US (31–49° N, 101–125° W) [5]. We adopt the ecoregion definition from Bailey *et al* (1994), and the spatial delineation of the western US from Yue *et al* [5, 30]. Ecoregions contain distinct habitats and historic fire regimes (figure S1).

For each year, climate scenario, and GCM, we calculated the quantity and spatial distribution of wildfire-related BC and OC emissions following the process described by Yue *et al*. We then calculated the total fuel load for each grid cell using the US. Forest Service × 1 km fuelbed map used by Yue *et al* (described further in McKenzie *et al* and Ottmar *et al*) [7, 31]. Similarly, fuel consumption rates for each fuel type were determined using table 4 in Spracklen *et al* [25] (included in the supplemental material), and assuming that fires burn with 25% of each high, medium, and low severity, and that 25% of the area remains unburned [32]. Total biomass consumed was then calculated as the product of total fuel load, applicable consumption rate, and area burned for the given grid cell for each month. Finally, BC and OC emissions were calculated by applying the biofuel burning emissions factors published by Andreae and Merlet [33]. Similar to Yue *et al* [5], we did not address potential effects of wildfires on the biosphere or the impact of climate change on vegetation type and extent. The resulting western US wildfire emission inventory contains monthly emissions of BC and OC simulated by five GCMs under two climate scenarios (RCP4.5 and RCP8.5) for each year in the 10 year periods centered on 2000, 2050, and 2090.

### 2.2. Wildfire-associated PM_2.5_ concentrations

We input our estimated wildfire BC and OC emissions for the western US, and non-wildfire anthropogenic emissions as indicated below, into the GEOS-Chem chemical transport model (version 12.6; http://acmg.seas.harvard.edu/geos/) to simulate impacts on PM_2.5_ concentrations, again repeating this process for each year, climate scenario, and GCM. We use the model at 0.5° × 0.625° resolution with 47 vertical levels (1013.25–0.01 hPa) over North America (60–130° W, 9.75–60° N). Boundary conditions for the nested North American domain are provided by a global simulation at 2° ×2.5° spatial resolution. We spin up the model for one month before any simulations to remove the effects of initial conditions.

Meteorological data were simulated with the Goddard Earth Observing System (GEOS-FP) from the NASA Modeling and Assimilation Office. Emissions of PM_2.5_ components over the US primarily included anthropogenic emissions from the National Emission Inventory 2011, and wildfire emissions from our inventory over the western US and the Global Fire Emissions Database version 4 over the eastern US [34]. To isolate the impacts of future western US wildfire emissions, we kept all meteorological data and emissions constant at the year 2000, except for our western wildfire emissions that corresponded to each simulation year in the 2000, 2050 and 2090 decades. Parallel work using similar methods and two of the five GCMs applied here has further established that climate change-induced changes in meteorological conditions could further increase PM_2.5_ [29].

We isolate the influence of climate-driven wildfire emission changes by comparing gridded PM_2.5_ concentrations simulated in the future years versus the present-day for each GCM separately. Our approach first simulates PM_2.5_ in the baseline excluding wildfire emissions; we then add wildfire emissions for the baseline period to simulate total baseline PM_2.5_; and finally we simulate future PM_2.5_ for each GCM/climate scenario combination for each of the two future eras (2050 and 2090), holding non-fire emissions constant at baseline levels. In this way we isolate the impact of wildfire emissions in both the baseline and future periods, and therefore discern the incremental impact of climate change on both future emissions and PM_2.5_.

### 2.3. Estimation of wildfire-associated PM_2.5_ health impacts

We estimate wildfire PM_2.5_-associated health impacts and economic values using the US EPA’s Environmental Benefits Mapping and Analysis Program—Community Edition (BenMAP—CE) version 1.5.1.0 [35, 36]. General forms of the epidemiological concentration-response functions used to calculate wildfire PM_2.5_-attributable health impacts are provided below:

(1a) $$y_{\text{ika}}={\text{Incidence}}_{\text{ika}}\times \left(1-{\text{e}}^{-\beta_{i}\times \text{WildfirePM}2.5_{k}}\right)\;\times \;{\text{Population}}_{ka}$$

(1b) $$y_{\text{ika}}={\text{Incidence}}_{\text{ika}}\times \left(1-\frac{1}{\left(1-{\text{Incidence}}_{\text{ika}}\right)\times {\text{e}}^{\beta_{i}\times \text{WildfirePM}2.5_{k}}+{\text{Incidence}}_{\text{ika}}}\right)\times {\text{Population}}_{ka}$$

(2) $$y_{i}=\underset{k}{\sum }\underset{a}{\sum }y_{\text{ika}\cdot }$$

For each health endpoint, the use of either the log-linear form (1a) or the logistic form (1b) is determined by the functional form in the epidemiological study used to derive the health impact function. In these equations, *i* signifies a specific health endpoint, *k* signifies a 0.5° × 0.625° GEOS-Chem grid cell in the western US, and *a* represents a population subgroup spanning 5 years of age. In equations (1a) and (1b), *y*_*ika*_ represents the wildfire-attributable PM_2.5_ health impacts for health endpoint *i* in grid cell *k* for population subgroup *a*; Incidence_*ika*_ is the baseline incidence of health endpoint *i* in grid cell *k* for population subgroup *a; β*_*i*_ is the effect estimate relating a change in PM_2.5_ concentration to a change in the risk of health impact *i*; WildfirePM2.5_*k*_ is the annual mean wildfire-attributable PM_2.5_ concentration in grid cell *k*, and Population_*ka*_ is the population subgroup in grid cell *k*. To calculate total wildfire attributable PM_2.5_ health impacts for a particular health endpoint during the baseline, 2050, or 2090 eras, health impacts are aggregated across all grid cells and all population subgroups as shown in equation (2). In equation (2), *y*_*i*_ signifies the total additional wildfire-attributable health impacts for a given health endpoint during the baseline, 2050, or 2090 eras when compared to the baseline without wildfires. We follow the US EPA methodology for estimating health risks from PM_2.5_ using concentration-response functions for total, not source-specific, PM_2.5_ mass. Thus, we apply total PM_2.5_ based health impact functions to the fine particles emitted as a result of wildfires; however, we limit our analysis of health impacts to those specific endpoints that have to date been associated with wildfire PM_2.5_ (listed in table 1).

For the baseline period, we use year 2000 population from BenMAP-CE. For the 2050 and 2090 eras, we use population projections derived for each 5 year age group at the county level using the EPA’s Integrated Climate and Land Use Scenarios v.2 (ICLUSv2) model [37, 38]. This ICLUSv2 scenario was developed as an intermediate growth population projection, with underlying assumptions about fertility, migration rate, and international immigration derived from the storyline for IPCC Shared Socioeconomic Pathway 2. County-level incidence rates were used as provided in BenMAP-CE for 5 year age groups and were originally derived from CDC WONDER [35]. We applied 2000 mortality rates for the baseline period, and matched the appropriate mortality rate projections for the 2050 era. As the CDC mortality rates were projected only through 2060, we apply 2060 mortality rates to the 2090 era analyses. Morbidity incidence rates are available only for 2014, so these rates are assumed to be constant. Results would scale approximately linearly with different projections of mortality rates.

### 2.4. Economic valuation of wildfire-associated PM_2.5_ health impacts

We estimate the economic value of projected health impacts based on recommendations in federal guidance for economic analyses [39] and valuation functions included in the BenMAP-CE model. These economic analyses consider the amounts that individuals are willing to pay for small reductions in mortality risk (‘value of statistical life (VSL)’) and the changes in VSL over time as real income grows (‘income elasticity’ of willingness to pay).

For mortality endpoints, the EPA’s Guidelines for Preparing Economic Analyses [39] recommends a VSL of $7.9 million (2008$) based on 1990 incomes. To create a VSL using 2015$ and based on 2010 incomes, the standard value was adjusted for inflation and income growth based on the approach described in EPA’s BenMAP-CE model and its documentation [35]. The resulting value, $9.7 million for 2010 (2015$), was adjusted to future years by assuming an income elasticity of VSL of 0.4 (since personal income projections are not available, gross domestic product (GDP) per capita is used as a proxy for mean personal income). Projections of US population change are described above, while the Emissions Predictions and Policy Analysis model [28] was run to generate a projection of GDP growth. Applying this standard approach yields VSL values of $12.4 million in 2050 and $15.2 million in 2090 (in undiscounted 2015$). As more recent literature favors a higher estimate of income elasticity of VSL, we also perform a VSL test using an elasticity of 1.0, reflecting proportional growth in VSL with GDP per capita. For morbidity endpoints, we use cost-of-illness estimates for each endpoint type available in BenMAP-CE. All morbidity endpoint estimates were adjusted from 2007$ to 2015$ using BenMAP-CE’s default inflation index.

## 3. Results

**Figure 2.**
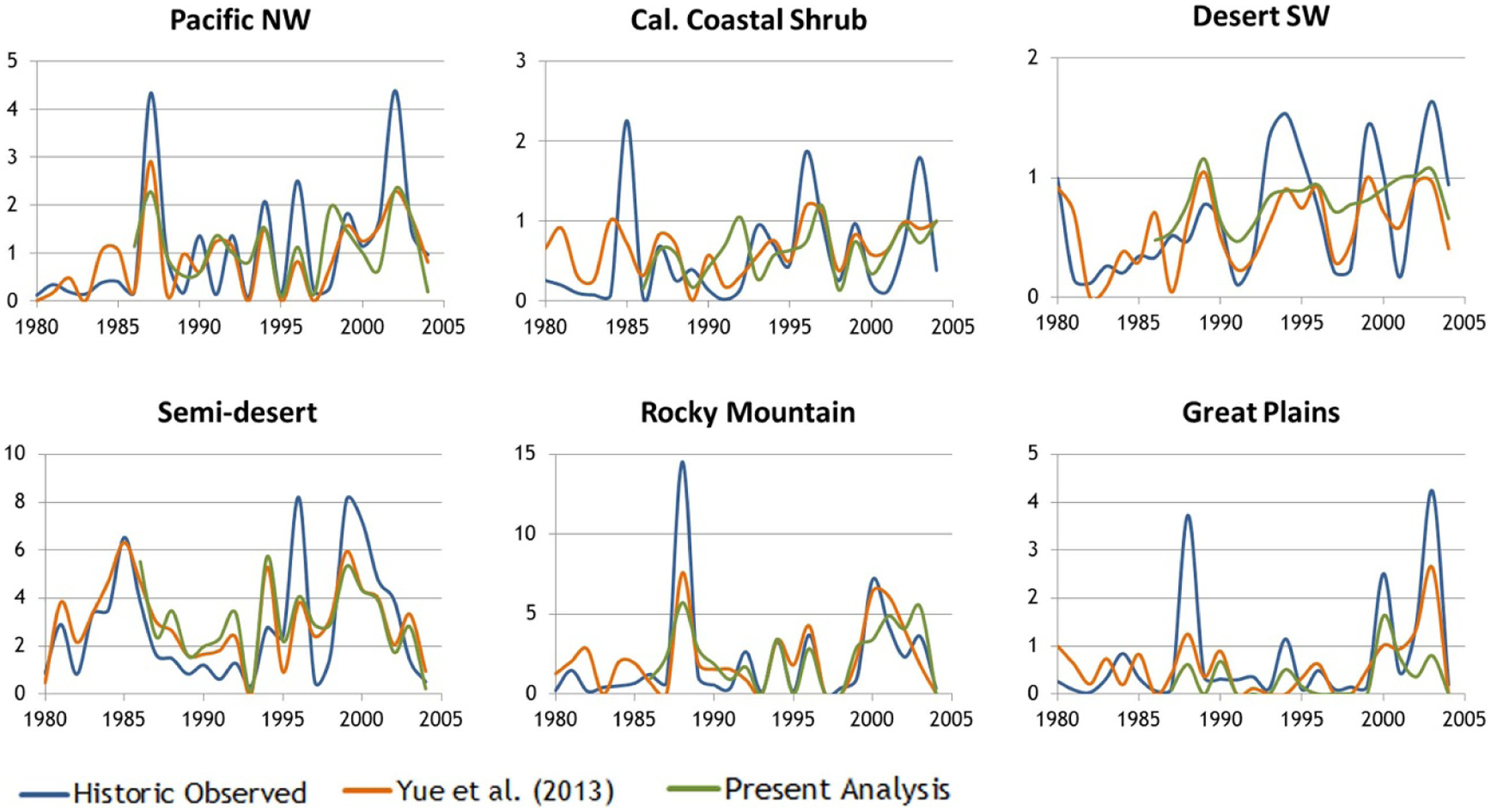
Summary of annual wildfire acres burned in historic observed, estimated by Yue *et al* (2013), and present analysis, for period 1980–2004 (100 000 ha). The 1980–2004 period provides the best overlap between the three time-series compared here. Supplemental material includes both a map of six ecoregions (figure S1), and analysis of model performance (see sections 2 and 3 and table S1).

**Figure 3.**
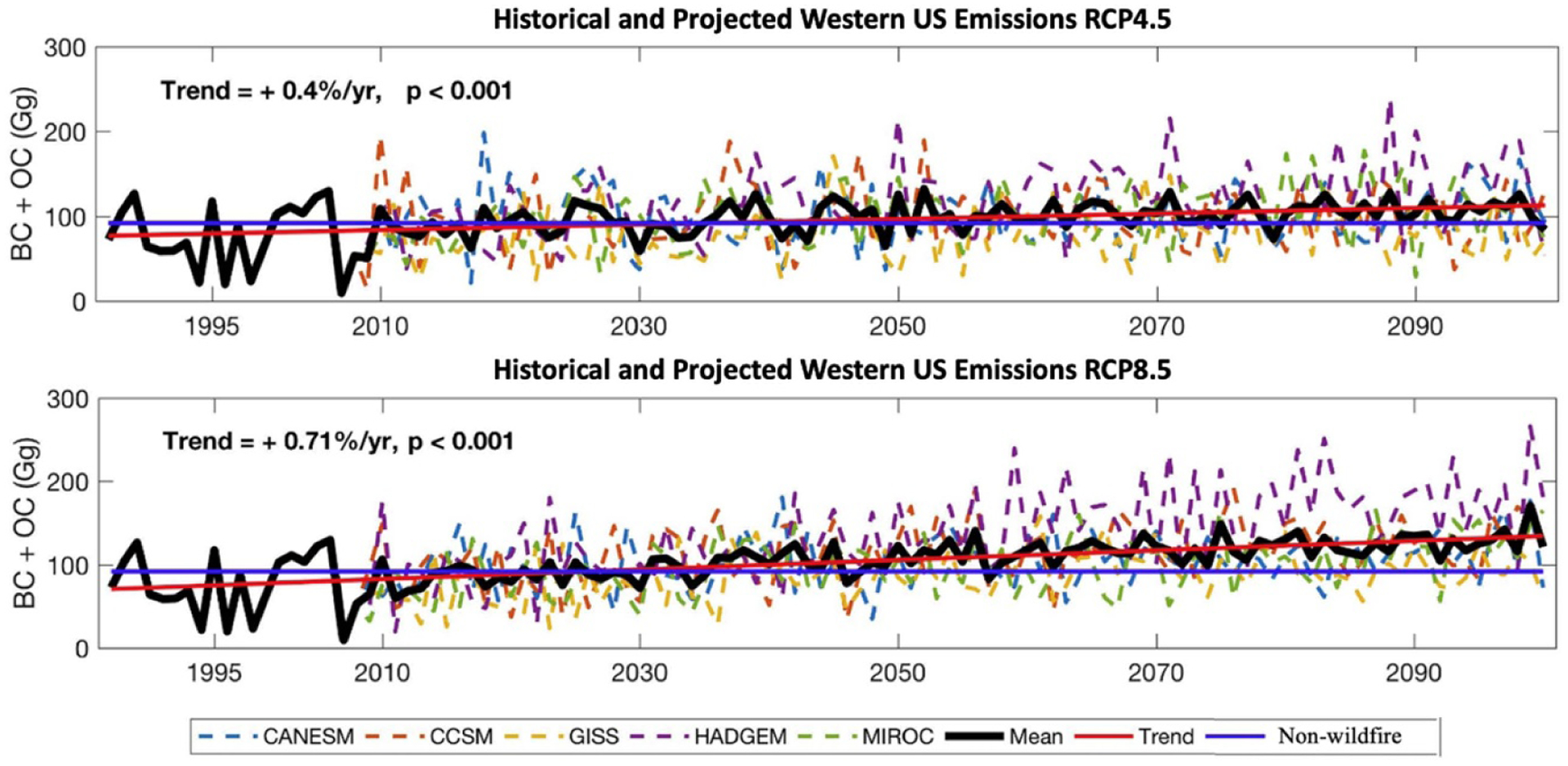
Time series of historical and projected estimated wildfire BC and OC emissions by RCP and GCM. Solid black line for mean is the historical emissions value through 2005, and the multi-GCM mean from 2006 to 2100. The red line (trend) is an ordinary least squares regression fit. Blue line is the non-wildfire emission input (labeled ‘Anth’ in the legend), which is held constant throughout the period and is provided for context. The supplemental material provides further detail on differences from baseline by GCM (table S4) and maps of BC and OC emissions by era, RCP, and GCM (figure S4).

Overall, our estimates of fire activity capture a substantial amount of interannual variability in area burned that can be attributed to climatic variation, though they generally underestimate peak years across all ecoregions (figure 2 and table S2). Underestimates of peak burn years are an important qualification of our results, particularly as western US burn area records set in the most recent 2020 fire season suggest the possibility of greater frequency of extreme annual wildfire incidence. We estimate that climatic factors increase wildfire BC and OC emissions under both RCP4.5 and 8.5, for all GCMs and future years (figure 3). Averaged across the five GCMs, BC and OC emissions increase 0.40% per year over the 2006–2100 period under RCP4.5 and 0.71% per year for RCP8.5 (*p* < 0.001). We find large interannual variability in wildfire BC and OC emissions driven by variability in precipitation and soil moisture inputs. Comparing between GCMs, we find that the estimated wildfire emissions increase is more moderate using simulated climate variables from a cooler climate model (GISS-E2-R), with virtually no change in emissions in both the 2050 and 2090 eras for RCP4.5, but increases of 8% in the 2050 era and 17% in the 2090 era relative to the 1996–2005 reference period for RCP8.5. The four other GCMs produce larger increases in emissions. HADGEM2-ES results have the largest magnitude change in emissions, with wildfire BC and OC emissions increasing from 2000 by over a third in the 2050 period for RCP4.5, and more than doubling for the 2090 period for RCP8.5. The results across GCMs largely correspond to variation in individual GCM projections of temperature and precipitation over the western U for our time period, and clearly illustrate the importance of using a wide range of plausible climate drivers when estimating future fire activity and emissions.

**Figure 4.**
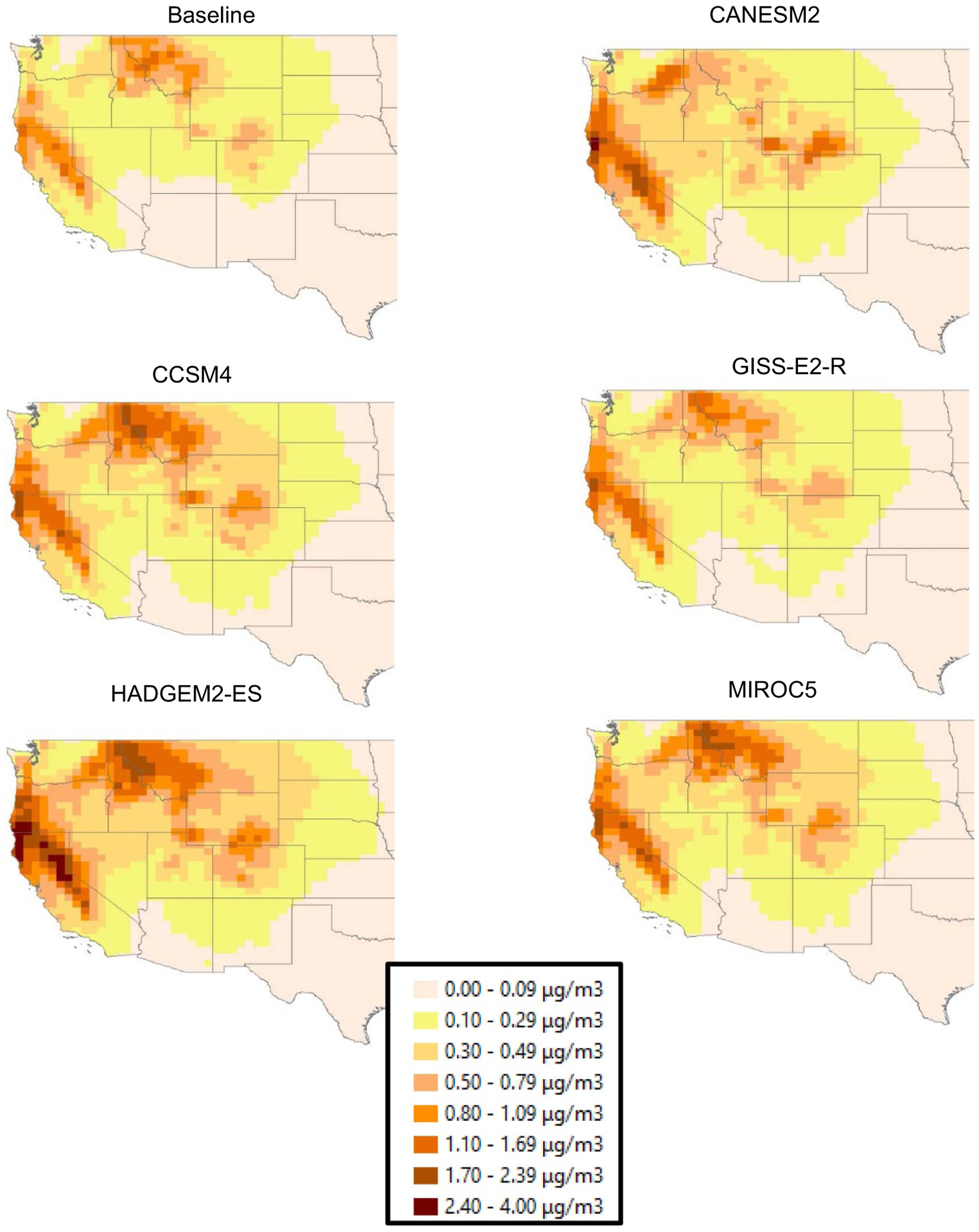
Wildfire-attributable annual average PM2.5 concentrations (*μ*g m^*−*3^) in the CONUS for RCP8.5 in the 2090 era. The estimates are the wildfire-attributable PM2.5 concentrations calculated by subtracting the no-wildfire from ‘with-wildfire’ simulated concentrations, for each GCM.

Figure 4 shows western wildfire-attributable PM_2.5_ concentrations for RCP8.5 in 2090 for all five GCMs. Spatially weighted PM_2.5_ concentrations from the CONUS GEOS-Chem results across all models, RCPs, and time periods are provided in the supplemental material. The HADGEM2-ES results show the largest impacts of wildfires, with a maximum change of nearly 4 *μ*g m^*−*3^ in 2090 relative to the baseline period. Compared to the baseline period value, western wildfire PM_2.5_ increases in most GCM/RCP/era combinations, more than doubling for the HADGEM2-ES/RCP8.5/2090 projection scenario. For all GCMs, the largest changes occur in the Nevada Mountain/Semi-Desert and Coastal California regions, and extend southward to Southern California along the Sierra Mountain range. Results in the supplemental material also show substantial differences between the RCP4.5 scenario (associated with lower temperatures, lower levels of wildfire area burned, lower BC and OC emissions, and lower PM_2.5_ concentrations) relative to RCP8.5, across nearly the entire western U.S (figure S6). GCMs other than HADGEM2-ES show less spatially uniform and smaller impacts of wildfires on PM_2.5_ concentrations, with the GISS-E2-R model showing the smallest increases from the present day.

These PM_2.5_ changes lead to impacts on mortality and morbidity across the CONUS. Results in table 1 show annual baseline wildfire PM_2.5_-attributable mortality and morbidity, and the annual excess PM_2.5_-attributable mortality and morbidity burden associated with future western US wildfires, averaged across the five GCMs. The estimates for mortality indicate that the excess burden in the 2090 RCP8.5 scenario is roughly 3.5 times larger than in the baseline period—however, there are 400 fewer premature mortality cases in RCP4.5 compared with RCP8.5 in 2090. For the RCP8.5 case, non-climate factors such as the projected increase in population, change in population composition by age, and projected reduction in baseline mortality rates over our simulation period together account for a large component of the change in health burden relative to baseline. Nonetheless about a 60% increase in mortality health burden in 2090 relative to baseline is attributable solely to the effect of climate change. Additional details are provided in the supplemental material (see table S9).

**Figure 5.**
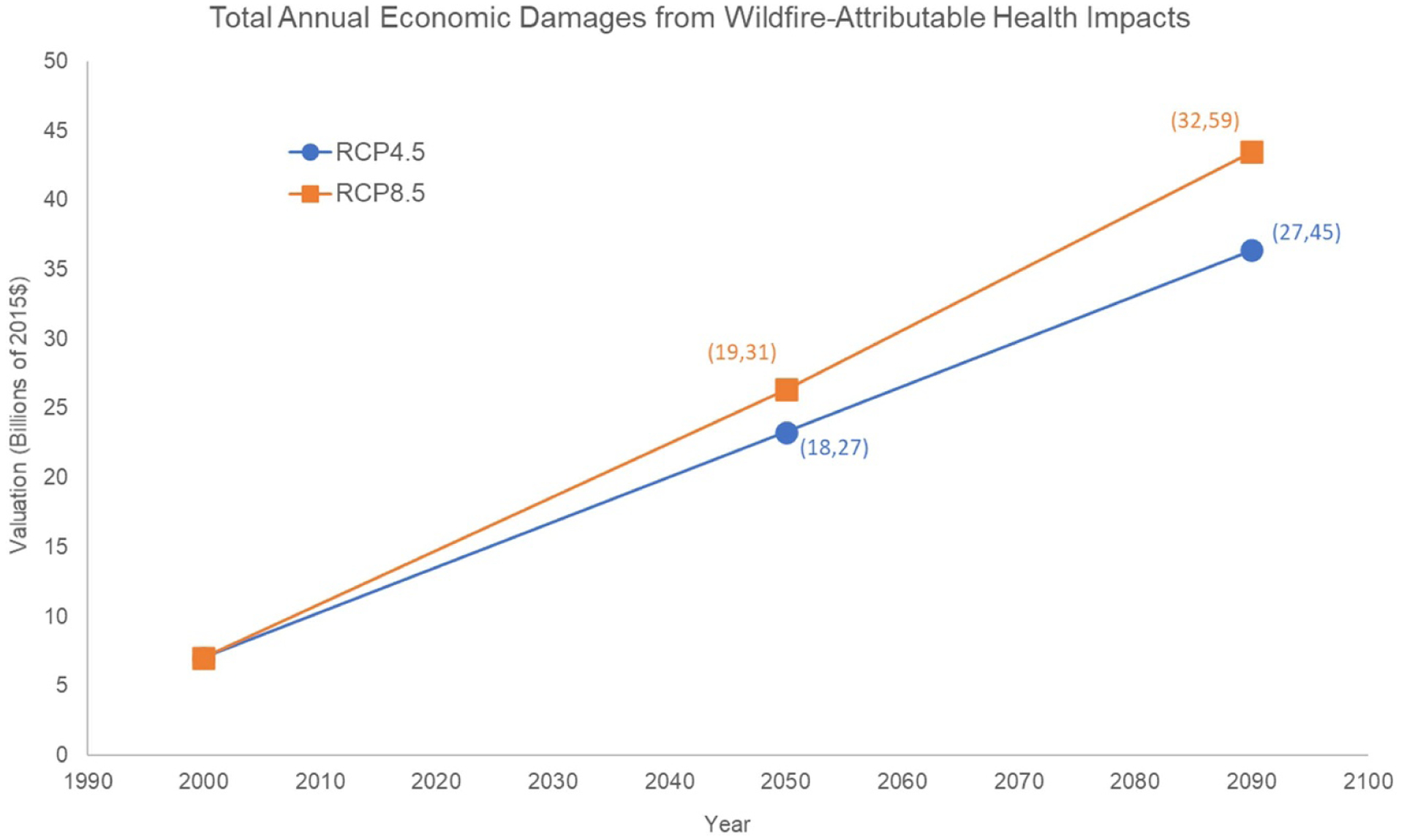
Summary of total annual economic damages from wildfire-attributable health impacts, for base period (2000) and two projection periods (2050 and 2090), for RCP 4.5 and 8.5 results, in billions of annual damages (2015$). Estimates are averages across runs for five GCMs, values in parentheses indicate lowest and highest GCM-specific estimate in the five-GCM ensemble.

Our estimates of the health and economic impacts depend in part on the effect of increased wildfire activity, but also the combined non-climate elements of the method, particularly population growth and increase in the valuation of avoided risk of premature mortality over time (associated with income growth). The overall result is a large increase in total health and health-related economic impact of wildfire-related PM_2.5_ mortality and morbidity, from roughly $7 billion per year in the baseline period to roughly $36 billion per year in 2090 for RCP4.5, and $43 billion per year in RCP8.5 (figure 5). The total economic burden of wildfire-attributable PM_2.5_ health impacts we are able to estimate steadily increases over time. As noted in the methods above, these estimates reflect use of an income elasticity for mortality valuation of 0.4—if we were to use an elasticity of 1.0 instead, estimated costs associated with the mortality burdens would be 87% higher in 2090, suggesting our valuation of the mortality endpoints may be conservative.

## 4. Discussion

Our results show the potential for a substantial increase in air pollution-related health impacts associated with exposure to PM_2.5_ from western US wildfires under plausible scenarios of future changes in climate. The increase is consistent across alternative projections of future climate and across multiple climate models, though the effect varies in magnitude and location across these futures. The increase in wildfire-related PM_2.5_ mortality and morbidity grows over time and is larger for the higher GHG scenario (RCP8.5) compared to the lower emission scenario (RCP4.5).

Our results are directly comparable to other estimates of climate change damages in the US that followed the CIRA framework, as we used the same climate scenarios, GCMs, and years of analysis. We estimated that future wildfire-attributable PM_2.5_-related mortality could increase by 3.7 times and 4.2 times in 2090 under RCP4.5 and RCP8.5, respectively, translating to $29 and $36 billion in annual total excess economic damages. An estimated 40% (for RCP4.5) and 60% (for RCP8.5) of the mortality increase is attributable to climate change, with the rest driven by population growth, change in population age structure, and mortality rate changes. Compared with other climate health impacts estimated in the CIRA framework, in 2090 under RCP8.5, these projected economic damages from increased wildfire PM_2.5_ mortality are larger than the climate-attributable impacts of West Nile Virus and hazardous algal blooms, smaller than those for extreme heat mortality and labor productivity, and comparable to those for southwestern US fugitive dust impacts (see comparison figure and full citations in the Executive Summary of US EPA 2017 [28]). For example, Achakulwisut *et al* [40] projected that damages related to dust-attributable cardiovascular mortality and asthma could increase from $13 billion/year historically to $47 billion/year by the end of the century, including future changes in population—comparable to the increase from a $7 billion baseline to a future $36 billion to $43 billion estimated here using the same climate scenarios, socioeconomic inputs, population scenarios, baseline health effects, and valuation estimates).

These findings are consistent with other studies in the literature, though they generally show a smaller impact of wildfires, which can be attributed to differences in analysis scope and methodology. Fann *et al* (2018) found that the baseline (2012) health burden for fire activity in the US is six times higher than our estimates, but that work includes the effect of prescribed fires, which predominate in the populous southeast region, and the effect of eastern U.S wildfires [9], both of which we exclude in our baseline and climate projections. Comparison of our estimates at a more granular level shows that, once we control for these differences in scope, our estimates show good agreement with those by Fann *et al* [9]. In addition, similar to our work but limited only to the PM_2.5_ concentration estimates, Li *et al* (2020) applied the GEOS-Chem model to western wildfires and found fine PM increases of 53% for RCP4.5 and a doubling under RCP8.5 by 2100.

Yue *et al* (2013) found higher estimates of both area burned and emissions (see figure 3)[5]. One reason is that our area burned regressions, based on bias-corrected GCM data, do not employ certain climate variables that Yue *et al* drew directly from GCM output without bias correction (e.g. wind speed is used by Yue *et al* but not in this work due to lack of data availability for spatially downscaled results from GCMs). Local scale estimates of wind speed could be important to area burned estimates (for example, Jin *et al* [4] find that about half of the area burned by wildfires in California coincide with Santa Ana winds[41]). However, it remains challenging to project local scale wind characteristics for mid- to late-21st century conditions from global climate models [42]. Another important reason that our results are lower than previous estimates is the spatial scale of analysis—our four times higher spatial resolution for area burned estimates (25 × 25 km versus 50 × 50 km) yields a lower overall mean annual area burned on net across the region (according to our sensitivity testing using a coarser grid). The effect of spatial scale of analysis is strongest for the California Coastal Shrub and Eastern Rocky Mountain regions.

Ford *et al* (2018) conducted a global scale analysis of wildfire impacts on PM_2.5_ that showed higher estimates of area burned, larger effects on PM_2.5_, and a larger health burden for the western US than results in our study [10]. We attribute the difference in results to four key differences in the methodology: (a) Ford *et al* use a single Earth system model (CESM) as opposed to our multi-model ensemble of five GCMs—CESM for the; (b) Ford *et al* use a coarser grid resolution (0.9° × 1.25°) similar to Yue *et al*; (3) we hold meteorology constant over time as an input to the GEOS-Chem air quality modeling, while Ford *et al* use time-varying meteorology; and (4) Ford *et al* consider changes in forest cover associated with forecast economic activity, which generally increases forest cover in the western US Although the combined directional effects of these methodological differences on results are unknown, in totality their effect is that our estimates are lower. The forecast changes in forest cover in Ford *et al*, a feature not included in our study, appears to have a large impact on their results. However, neither Ford *et al* nor we consider changes in vegetation type. Some wildfire studies using dynamic vegetation models [28] suggest that historically-dominant plant species in many Western areas are replaced with those having less-frequent fire return intervals. These shifts can result in lower levels of future burning than would be anticipated in an analysis that does not include these dynamic changes [7].

Regarding time-varying meteorology, in our study running GEOS-Chem with future meteorology was not possible since the model was not configured to use outputs from global climate models. Recent evidence suggests, however, that our use of present-day meteorological fields when simulating wildfire emissions effects on PM_2.5_ concentrations likely leads to underestimated impacts on wildfire-related PM_2.5_ due to a shorter lifetime of PM_2.5_ than might be expected in the future in areas projected to experience reductions in precipitation [29, 43, 44]. The net effects of climate change on meteorology remain important for estimating PM_2.5_ concentrations. Results of a parallel effort using a different air quality model, but otherwise similar methods and inputs with the exception of climate-driven wildfire emissions, show that changes in meteorology alone could increase population-weighted PM_2.5_ exposure in CONUS by 0.08–0.18 *μ*g m^*−*3^ by mid-century, and 0.13–0.65 μg m^*−*3^ by end century, leading to an additional 15 000–21 000 annual PM_2.5_-attributable premature deaths by the end of the century [29]. Other literature suggests the effect of changes in future meteorology on PM_2.5_ concentrations may remain uncertain or at least highly context-specific, so future research that integrates forecast meteorology with changes in wildfire emissions attributable to climate change remains a high priority [45].

There are also other reasons to suggest that our results underestimate the full air pollution-related health burden of wildfire. We focus on the annual average concentration of PM_2.5_, but increasingly the mixture of air pollutants that constitute wildfire smoke has been implicated in health burden, particularly short-term effects associated with pollutants other than PM_2.5_ (e.g. air toxics) [21, 23]. In addition, the overall economic burden of wildfire clearly extends beyond the health impact. The most well documented costs are those reported right after a major fire and include the direct costs of fire suppression and firefighting, as well as evacuations, equipment damage, damaged property, school and business closures, and short-term public health alerts (Association for Fire Ecology, 2015). The immediate annual and cumulative wildfire response costs alone (excluding property damage and business closures) are roughly proportional to acres burned, and for our study would represent an additional excess annual economic burden of $640 million to $730 million in 2050, and $710 million to $890 million in 2090 (see calculations in supplemental material). These costs fail to include more nuanced and long-lasting impacts that wildfires can have on things as far reaching as public water supplies and the performance of photovoltaic installations [46–48]. In total, Thomas *et al* (2017) find that the annualized economic burden from wildfire is between $71.1 billion to $347.8 billion (2016 US$) [49]. Table 2 provides a summary assessment of some of the key assumptions involved in this complex multi-disciplinary, multi-model study, and the potential impact of the assumption on the overall estimates. As noted in the third column, if information exists to assess the assumptions, in most cases the impact is to underestimate impacts. Improving the basis and ability to conduct quantitative uncertainty and sensitivity analysis for these assumptions in particular represent important areas for future research.

## 5. Conclusion

We demonstrate that climatic change over the 21st century has the potential to greatly increase the overall air pollution-related health burden of western wildfires. Our work extends and complements prior work in this area. In particular, a key contribution of our work to this literature is providing detailed estimates of the impact of wildfire PM_2.5_ on premature mortality, and various forms of morbidity, in communities near the source of wildfire smoke but also extending to areas far downwind (recently evidenced in the 2020 wildfire season [50]), using more highly resolved emissions and air quality estimation tools than prior work, and for a strategically chosen wide range of climate projections (five GCMs and two RCPs).

## Supplementary Material

Supplementary Material

## Figures and Tables

**Table 1.** Wildfire PM_2.5_-attributable health burden (mortality and morbidity)—total incidence burden (cases per year) for base period (1996–2005) and projected excess health burden associated with climate-induced changes in wildfire PM_2.5_ for 2050 and 2090 eras and each RCP scenario. Excess burden is calculated as the average of wildfire PM_2.5_-attributable health burden estimates across all five GCMs, in excess of the reference burden. Range of results across GCMs is provided in parentheses below the mean estimate.

				Ten year averaged excess burden relative to reference (cases per year)	
Health endpoint	Age (years)	Reference burden (cases per year)	Future climate scenario	2050 cases (range across GCMs)	2090 cases (range across GCMs)
Mortality, all cause (long-term, based on Krewski *et al*)	30–99	720	RCP4.5	1300 (880, 1600)	1900 (1300,2500)
			RCP8.5	1600 (980, 2000)	2300 (1600, 3300)
Acute myocardial infarction, nonfatal	18–99	500	RCP4.5	1000 (740, 1300)	1500 (1100, 1900)
			RCP8.5	1200 (820, 1500)	1800 (1300, 2600)
Hospital admissions, all types	0–99	730	RCP4.5	1500 (1000, 1800)	2200 (1500,2800)
			RCP8.5	1800 (1200, 2200)	2600 (1900, 3700)
Emergency department visits, asthma	0–99	400	RCP4.5	310 (160,410)	480 (280,670)
			RCP8.5	390 (200, 530)	620 (380,940)
Acute bronchitis	8–12	1300	RCP4.5	770 (340, 1100)	1300 (680, 1800)
			RCP8.5	1000 (440, 1400)	1600 (950, 2600)
Upper and lower respiratory symptoms	9–11	41 000	RCP4.5	23 000 (10 000, 32 000)	38 000 (20 000, 55 000)
			RCP8.5	31 000 (14 000, 43 000)	50 000 (29 000, 78 000)
Asthma exacerbation: cough, shortness of breath, and wheeze	6–18	98 000	RCP4.5	71 000 (24 000, 120 000)	113 000 (49 000, 200 000)
			RCP8.5	110 000 (32 000, 180 000)	160 000 (70 000, 330 000)
Work loss days	18–64	100 000	RCP4.5	57 000 (24 000, 81000)	98 000 (52 000, 140 000)
			RCP8.5	77 000 (32 000, 110 000)	130 000 (74 000, 200 000)
Minor restricted activity days	18–64	610 000	RCP4.5	340 000 (150 000, 480 000)	580 000 (320 000, 830 000)
			RCP8.5	460 000 (200 000, 640 000)	760 000 (440 000, 1200 000)

**Table 2.** Summary of key assumptions and estimated effect on overall results.

Key assumption	Analytic step	Comments and estimate of direction of potential bias in health burden impact estimates
Use of regressions to estimate area burned	Calculation of area burned	Likely underestimate, potentially major. Evaluation of regression performance for historical data finds underestimate of extreme years of area burned. The result is likely an underestimation of total impacts
Regressions omit effects of wildfire and climate change on vegetation type and extent	Calculation of area burned and conversion to emissions	Unknown impact. Wildfire will lead to loss of biomass followed by regeneration of vegetation but with unknown timing. Climate change is expected to change vegetation type and extent but in uncertain ways.
Spatial and temporal disaggregation of area burned based on historical patterns	Calculation of area burned	Unknown impact. Future spatial pattern of area burned is uncertain.
Use of US Forest Service fuel type dataset to estimate fuel quantity in each grid cell	Conversion of area burned to emissions	Unknown impact. Future fuel quantities could be higher in grid cells if climate change increases biomass, or lower if frequent burns reduce biomass in that grid cell
Use of constant BC and OC emissions factors throughout study period	Conversion of area burned to emissions	Unknown impact, likely minor. There is not believed to be an inherent bias in emissions factors over time, but the overall impact of this assumption is likely less important than fuel quantity
Use of present-day meteorology throughout study period	Simulation of ambient pollutant concentrations	Likely underestimate, potentially major New research (Fann *et al* 2021) suggests that future meteorology could present a major ‘climate penalty’ for PM_2.5_ concentrations, but this factor has not yet been fully integrated with an assessment of future climate-induced wildfire risk
Omission of potential for secondary aerosol formation from wildfire nitrogen oxide emissions	Simulation of ambient pollutant concentrations	Likely underestimate, impact may be minor. While nitrogen oxide emissions result from wildfires, and can lead to secondary aerosol formation, the good agreement of our historical simulations with observed PM_2.5_ concentrations suggests the impact is likely to be small.
Omission of effects of wildfire emissions mixtures	Simulation of health burden	Likely underestimate, unknown impact. Characterizing health effects from exposures to complex wildfire emissions mixtures require further study.
Economic valuation focuses only on health impact, and is based on conservative assumptions about the income elasticity of willingness to pay to avoid mortality risk	Estimation of economic burden	Likely underestimate, potentially major impact. Wildfire has a wide range of economic impacts, including property damage, business interruption, and response costs. Only a portion of these impacts are addressed here, and some likely large impacts such as property damage have only been assessed historically or for limited contexts. This study also uses EPA’s standard estimate of 0.4 for income elasticity, while more recent reviews suggest that an estimate of 1.0 may be justified, which could increase estimates for 2090 by over 80%.
