# Supplementary material for: Estimating PM2.5-related premature mortality and morbidity associated with future wildfire emissions in the western US
Source: Environ Res Lett. Author manuscript; Available in PMC 2021 Apr 15. (PMC8048092; doi:10.1088/1748-9326/abe82b)
Supplement: Supplementary Material [file NIHMS1682309-supplement-Supplementary_Material.pdf]

Supplemental Material for:

Estimating PM2.5-related premature mortality and morbidity associated with future wildfire emissions in the Western U.S.

James E. Neumann, Meredith Amend, Susan Anenberg, Patrick L. Kinney, Marcus Sarofim, Jeremy Martinich, Julia Lukens, Jun-wei Xu, and Henry Roman

Contents:

1. Data Documentation, Sources, and Availability
2. Overall Methodological and Area Burned Approach
3. Relative Humidity and Rocky Mountain Ecoregion Regression
4. LOCA-based Regression Model Performance Against Historic Data
5. Additional Details on Emissions Calculations
6. GEOS-Chem Air Quality Results
7. BenMAP Modeling and Detailed Health Burden Results
8. Wildfire Response Costs

1. Data Documentation, Sources, and Availability

Comprehensive technical documentation describing the second phase of the Climate change Impacts and Risk Analysis (CIRA2.0) modeling framework, inputs, and limitations is publicly available (USEPA 2017). Each combination of the two RCPs and five GCMs used in this work was downscaled from the native GCM spatial resolution to a 1/16 degree latitude and longitude scale (an approximately 6.25 km grid) over the contiguous US (USBR et al. 2016). The statistical downscaling technique, LOCA (LOcalized Constructed Analogs), uses a multi-scale spatial matching scheme to pick appropriate analog days from observations. The LOCA dataset provides daily projections through 2100 for three variables: daily maximum temperature, daily minimum temperature, and daily precipitation (see USBR et al. 2016 for more details).

Population projection data relies on US national estimates under the Median Variant Projection (UN 2015), and county-level population projections derived using the Integrated Climate and Land Use Scenarios version 2 (ICLUSv2) model (Bierwagen et al. 2010; USEPA 2017b). The spatial pattern of population change in ICLUSv2 relies on assumptions regarding fertility, migration rate, and international immigration – these were parameterized based on the Shared Socioeconomic Pathway (SSP) 2, which suggests medium levels of fertility, mortality, and international immigration (O'Neill et al. 2014).[1] Where necessary for economic valuation, we used the Emissions Predictions and Policy Analysis (EPPA, version 6; Chen et al. 2015) model was run to generate a projection of economic growth (i.e., gross domestic product, or GDP).

[1] O'Neill, B. C., E. Kriegler, K. Riahi, K. L. Ebi, S. Hallegatte, T. R. Carter, R. Mathur, and D. P. v. Vuuren. 2014. A new scenario framework for climate change research: the concept of shared socioeconomic pathways, *Climatic Change*, doi:10.1007/s10584-013-0905-2.

Data Type	Description	Data Documentation and Availability
**a. Data Used in this Study**		
Bias-corrected and downscaled temperature and precipitation projections	Localized Constructed Analogs (LOCA) contain daily temperature (max and min) and precipitation data for a range of CMIP5 climate scenarios, baseline, and projection years.	U.S. Bureau of Reclamation, Climate Analytics Group, Climate Central, Lawrence Livermore National Laboratory, Santa Clara University, Scripps Institution of Oceanography, U.S. Army Corps of Engineers, and U.S. Geological Survey, 2016: Downscaled CMIP3 and CMIP5 Climate Projections: Release of Downscaled CMIP5 Climate Projections, Comparison with Preceding Information, and Summary of User Needs. Data available at: http://gdo-dcp.ucllnl.org/downscaled_cmip_projections/.
Observed meteorology	Historical climate data for temperature, precipitation, and other weather variables.<br><br>Reference to spatial coverages used in the Yue et al. (2013) work were used to ensure proper replication of the area burned calculations from that study. These rely on NOAA GSOD and USHCN datasets.	Livneh, B., et al. A spatially comprehensive, hydrometeorological data set for Mexico, the U.S., and Southern Canada 1950-2013. **Scientific Data** 2, 150042 (2015). Available online at: https://data.nodc.noaa.gov/cgi-bin/iso?id=gov.noaa.nodc:0129374<br>Sheffield, J., G. Goteti, and E. F. Wood, 2006: Development of a 50-yr high-resolution global dataset of meteorological forcings for land surface modeling. *J. Climate*, **19**, 3088-3111 Global Meteorological Forcing Dataset for Land Surface Modeling. Available online at: http://hydrology.princeton.edu/data.pgf.php<br>NOAA Global Surface Summary of the Day (GSOD) data available at: https://data.nodc.noaa.gov/cgi-bin/iso?id=gov.noaa.ncdc:C00516<br>NOAA United States Historical Climatology Network (USHCN) data available at: https://www.ncdc.noaa.gov/data-access/land-based-station-data/land-based-datasets/us-historical-climatology-network-ushcn
Ecoregion designations for fire emissions modeling	Ecoregion delineations were adapted from Bailey et al. (1994) to ensure consistency with LOCA data – see Figure S1 below and accompanying text below.	Bailey, R., P. Avers, T. King, and W. McNab (1994), Ecoregions and subregions of the United States (map), technical report, USDA For. Serv., Washington, D. C.
Fuelbed and Western wildfire emissions data	Biomass consumption by wildfire estimated using results of Spracklen et al. (2009) and biofuel burning emissions factors from Andreae and Merlet (2001).	Spracklen, D. V., Mickley, L. J., Logan, J. A., Hudman, R. C., Yevich, R., Flannigan, M. D., & Westerling, A. L. (2009). Impacts of climate change from 2000 to 2050 on wildfire activity and carbonaceous aerosol concentrations in the western United States. *Journal of Geophysical Research: Atmospheres*, 114(D20).<br>Andreae, M. O., & Merlet, P. (2001). Emission of trace gases and aerosols from biomass burning. *Global biogeochemical cycles*, 15(4), 955-966.
Baseline non-Western wildfire emissions	Eastern U.S. fire emissions from GFED4 dataset; Eastern and Western U.S. non-wildfire and anthropogenic emissions from USEPA National Emissions Inventory (NEI) 2011 data.	Global Fire Emissions Dataset (GFED) available at: https://www.globalfiredata.org/index.html<br>USEPA National Emissions Inventory (NEI) data available at: https://www.epa.gov/air-emissions-inventories/national-emissions-inventory-nei

Data Type	Description	Data Documentation and Availability
Atmospheric modeling	Open access GEOS-Chem model version 12.5.0 (September 2019).	Model and documentation available at: http://acmg.seas.harvard.edu/geos/
Baseline health effect incidence rates	County-level baseline incidence rates were obtained from BenMAP-CE v.1.4.14 for five-year age groups and were originally derived from CDC WONDER (U.S. EPA, 2018b). CDC mortality rates were projected only through 2060, so we apply 2060 mortality rates to the 2090 era analyses. Baseline morbidity rates are available only for 2014, so these rates are assumed constant across all eras.	U.S. EPA. (2018a). *Environmental Benefits Mapping and Analysis Program: Community Edition (BenMAP-CE) User Manual and Appendices*. Washington, DC.<br>U.S. EPA. (2018b). *Environmental Benefits Mapping and Analysis Program: Community Edition (BenMAP-CE) v 1.4.14*. Washington, DC.<br><br>Baseline incidence data presented in in tables below.
Population and developed land projections	Median Variant Projection of the United Nation's (UN) 2015 *World Population Prospects* dataset used to project future U.S. population for 2015-2100.	United Nations, 2015: World Population Prospects: The 2015 Revision. United Nations, Department of Economic and Social Affairs, Population Division. Data available at: https://population.un.org/wpp/
	U.S. national and county-level population figures from 2000-2015	U.S. Census Bureau, cited 2017: Population Estimates Program. Available online at https://www.census.gov/programs-surveys/popest.html
	County-scale population and developed land projections from the Integrated Climate and Land-Use Scenarios model (version 2)	Population projection documentation available at this link https://www.epa.gov/iclus<br>EPA, 2017: Updates to the Demographic and Spatial Allocation Models to Produce Integrated Climate and Land Use Scenarios (ICLUS) (Version 2). U.S. Environmental Protection Agency, Washington, DC, EPA/600/R-16/366F. Available online at https://cfpub.epa.gov/ncea/iclus/recordisplay.cfm?deid=322479
Domestic economic growth	Projection of future gross domestic product from the Emissions Predictions and Policy Analysis (EPPA, v6) model.<br><br>The projection of GDP growth through 2040 from the 2016 Annual Energy Outlook reference case is used to calibrate EPPA-6, and is also	Chen, Y.-H. H., et al. The MIT EPPA6 Model: Economic Growth, Energy Use, and Food Consumption. MIT Joint Program on the Science and Policy of Global Change, Report 278, Cambridge, MA (2015)<br><br>U.S. Energy Information Administration, 2016: Annual Energy Outlook. Available online at https://www.eia.gov/outlooks/archive/aeo16/

Data Type	Description	Data Documentation and Availability
	then combined with EPPA-6 baseline assumptions for other regions and time periods	
Economic valuation of health burden estimates	Economic value of projected health impacts based on recommendations in federal guidance for economic analyses (U.S. EPA, 2014) and valuation functions included in the BenMAP-CE model. For mortality endpoints, the EPA's Guidelines for Preparing Economic Analyses recommends a VSL of $7.9 million (2008$) based on 1990 incomes (U.S. EPA, 2014).<br>For morbidity endpoints, we use cost-of-illness estimates for each endpoint type available in BenMAP-CE.	U.S. EPA. (2018a). *Environmental Benefits Mapping and Analysis Program: Community Edition (BenMAP-CE) User Manual and Appendices*. Washington, DC.<br>U.S. EPA. (2018b). *Environmental Benefits Mapping and Analysis Program: Community Edition (BenMAP-CE) v 1.4.14*. Washington, DC.<br><br>To create a VSL using 2015$ and based on 2010 incomes, the standard value was adjusted for inflation and income growth based on the approach described in EPA's BenMAP-CE model and its documentation (U.S. EPA, 2018a,b). The resulting value, $9.7 million for 2010 (2015$), was adjusted to future years by assuming an income (GDP per capita) elasticity of VSL of 0.4. Applying this standard approach yields the following VSL values: $12.4 million in 2050, and $15.2 million in 2090 (in undiscounted 2015$).
Price deflator	Dollar years are adjusted to $2015 using the U.S. Bureau of Economic Affairs' Implicit Price Deflators for Gross Domestic Product, Table 1.1.9.	U.S. Bureau of Economic Affairs' Implicit Price Deflators for Gross Domestic Product, Table 1.1.9. See "National Income and Product Accounts Tables" at https://bea.gov/national/index.htm
**b. Data Produced by This Study**		
Full results and analysis code	Shape files for LOCA 1 x 1 degree grid.<br><br>Shape files with area burned and emissions results.<br><br>Shape files with GEOS-Chem modeling results<br><br>BenMAP-CE configuration and results files	Data will be posted to ScienceHub and EPA's Environmental Dataset Gateway (EDG) upon publication of the manuscript.

2. Overall Methodological Approach and Area Burned Analyses

The five climate models applied here were from the Canadian Centre for Climate Modeling and Analysis (CanESM2, Von Salzen et al.2013); the National Center for Atmospheric Research (CCSM4, Gent et al. 2011, Neale et al. 2013); the NASA Goddard Institute for Space Studies (GISS-E2-R, Schmidt et al. 2006); the Meteorological Office at the Hadley Centre (HadGEM2-ES, Collins et al., 2011; Davies et al. 2005); and the Atmosphere and Ocean Research Institute, National Institute for Environmental Studies, and Japan Agency for Marine-Earth Science and Technology (MIROC5, Watanabe et al. 2010).

As the GCMs have very coarse spatial resolution, we use spatially downscaled climatic variables from the LOcalized Constructed Analogs (LOCA) dataset for each RCP and GCM combination. The LOCA dataset provides daily projections through 2100 at a 1/16 degree resolution (~6.25 km) for three variables: daily maximum temperature (tmax), daily minimum temperature (tmin), and daily precipitation (see U.S. EPA 2017a for more details).

It is important to place wildfire activity that occurs in the western U.S. (31°–49°N, 101°–125°W) in the context of total wildfire activity across the contiguous U.S. As part of the Monitoring Trends in Burn Severity (MTBS) project, the USGS Center for Earth Resources Observation and Science (EROS) and the USDA-FS Remote Sensing Applications Center compiled fire data from federal agency databases (ICS 209) and state databases, then used Landsat remotely-sensed imagery data to map burn severity and perimeters of all large fires in the U.S. from 1984 to 2016. This dataset is limited by its exclusion of small fires (fires smaller than 500 acres in the east and smaller than 1,000 acres in the west). However, even with the exclusion of smaller fires, the dataset captured over 95% of area burned when compared to ground-based data in 2004. Hence, the MTBS data is used to estimate the fraction of contiguous U.S. wildfire activity that has historically occurred in the western U.S., both with respect to number of fires and area burned.

Prescribed wildfires and unknown wildfire types were excluded from the dataset before calculations spanning the baseline years (1986-2005) were performed. With respect to the number of fires, on average, there were 2.4 times as many fires in the West than in the East from 1986-2005. Put differently, 70% of fires in the contiguous U.S. occurred in the western U.S. on an average annual basis. The differential size thresholds for inclusion in the MTBS dataset result in an underestimate of contributions of Western fires, given that Western fires must be double the size of Eastern fires to be counted. Thus, not only does the West have a higher number of fires than the East, but the West also has larger fires. Accordingly, there were 5.6 times as many acres burned in the West than in the East on average, and 81% of area burned was located in the Western U.S. on an average annual basis from 1986-2005. In effect, this analysis captures a large fraction of contiguous U.S. wildfire activity in examining the western U.S.

The present analysis builds upon existing research relating climatic conditions to annual area burned to estimate the future health burden associated with wildfire emissions in the western U.S. The flow chart in main text Figure 1 summarizes our approach and highlights the components that are unique to the present analysis and those that have been adopted from earlier

research. In this supplement we have reproduced key inputs from Yue et al., (2013), Spracklen et al. (2009) and Andre and Merlet (2001) that were relied on for this analysis.

The spatial and temporal disaggregation described in step 3 of main text Figure 1 was adopted from Yue et al., (2013). As part of that process, the annual area burned in each ecoregion is distributed across the landscape and each month of the wildfire season while maintaining historic patterns of wildfire activity. Table S3 from Yue et al. (2013) (found here: https://www.ncbi.nlm.nih.gov/pmc/articles/PMC3763857/#FN3) provides the historic burn fraction for each ecosystem that was used as part of the spatial disaggregation process.

Yue et al. (2013) presents two alternative methodologies for relating climate variables to wildfire area burned in the western U.S. The first approach expands on earlier work conducted by Spracklen et al. (2009) and utilizes a stepwise regression analysis to relate observed annual area burned in each ecoregion to several meteorological predictor variables and fire indexes. The second approach is a physical parameterization of area burned based on monthly mean temperature, relative humidity and precipitation. The two approaches differ not only with respect to the specific climate variables utilized but also by their temporal resolution – the regression models predict annual area burned, while the parameterization estimates monthly area burned.

Here, we use the six ecoregion-specific regression models because their predictions of present-day area burned are more consistent with observations than the parameterized model in every ecoregion other than the Great Plains. We modified and re-estimated the regression model for Rocky Mountain Forest ecoregion, as described in the section 3 below – the result is summarized in Table S1. The regressions and R^2 statistics for five of the ecoregions (Pacific Northwest, California Coastal Shrub, Desert Southwest, Nevada Mnt./Semi-Desert, and Eastern Rocky Mtn./Great Plains)_are used directly from Yue et al., 2013, and are available in Table 1 here: https://www.ncbi.nlm.nih.gov/pmc/articles/PMC3763857/#FN3). Of particular importance is that the regression models perform better than prior work in the Pacific Northwest and California Coastal Shrub ecoregions. These two ecoregions contain a large percentage of total population in the western United States and are therefore expected to comprise a significant proportion of the future health impacts associated with wildfire smoke.

Table S1. Modified wildfire regression model for the Rocky Mountain Forest ecoregion

Ecoregion	Regression Model[a]	R^2
Rocky Mtn. Forest	-9.4×10^5 *(Precipitation during Fire Season) - 13.5×10^5	45%

Overall, our regressions capture a substantial amount of interannual variability in area burned that can be attributed to climatic variation, though they generally underestimate peak years across all ecoregions (Main Text Figure 2 and Table S2). The largest differences are observed for the Great Plains region, where we underestimate area burned by a factor of about 2.7. Model performance is much better for the Nevada Mountains, Rocky Mountain Forest, Pacific Northwest, and Desert

Southwest, and California Coastal regions, which account for most of historical area burned and dry biomass consumption.

The climate variables and fire indexes required for each regression model were calculated using daily LOCA data, consistent with other CIRA analyses. To be consistent with Yue et al. (2013) models, only LOCA grid cells that intersect with the meteorological observations used in Yue et al. (2013) were used (Fig. S1), These meteorological observations include the Global Surface Summary of the Day (GSOD) sites or United States Historical Climatology Network (USHCN) sites, depending on the type of climate variable – temperature and precipitation variables were calculated from USHCN sites and relative humidity variables and fire indexes were calculated from GSOD sites.

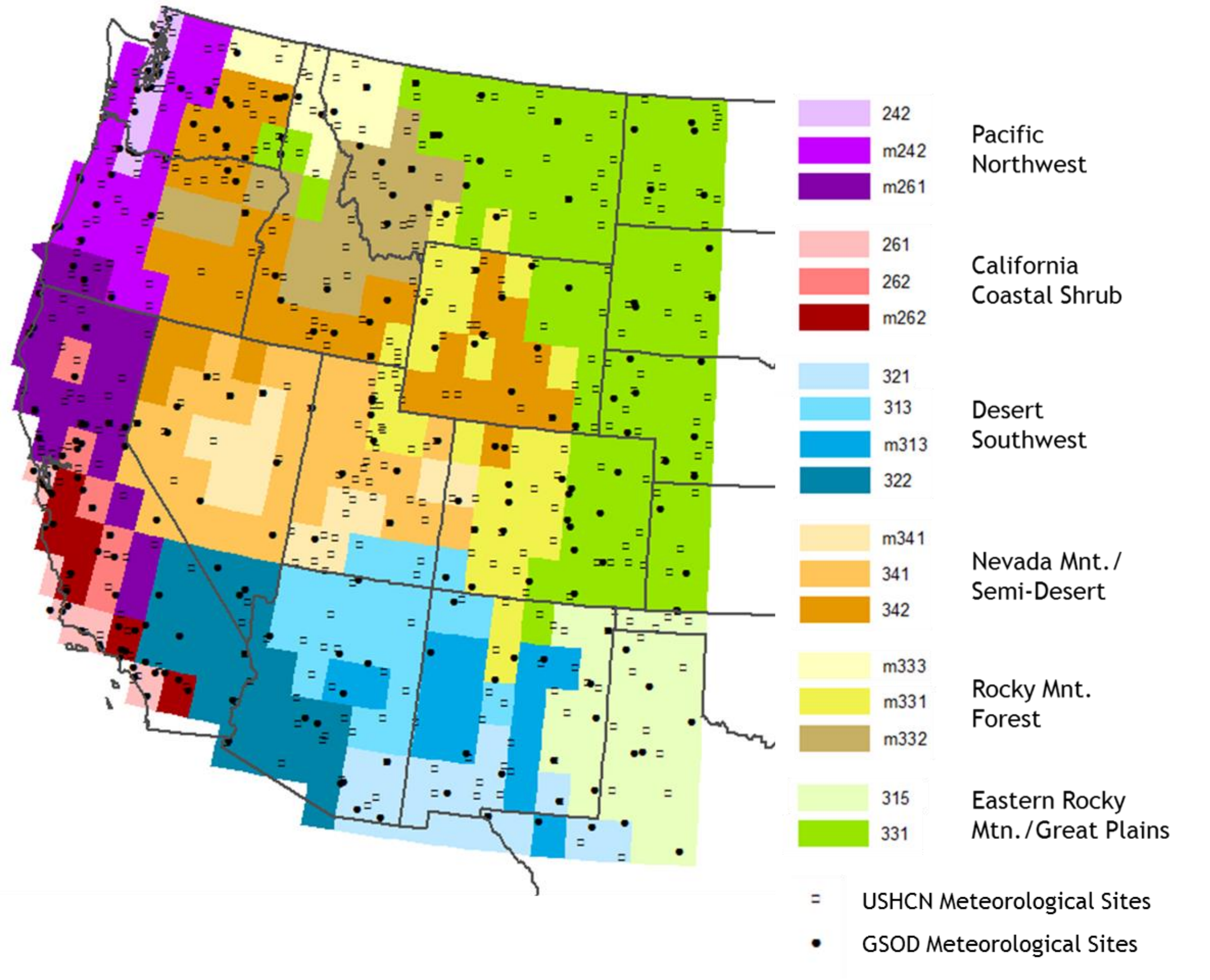

Figure S1. Western U.S. ecoregions and ecosystems (map generated from descriptions and contour mapping from Bailey et al., 1994, translated to analytic grid form and augmented with meteorological site locations by the authors). The color shading within each ecoregion is related to the historic burn fraction of each ecosystem (i.e., ecosystems with a larger fraction of area burned are represented with darker colors). The United States Historical Climatological Network

(USHCN) and Global Surface Summary of the Day (GSOD) meteorological sites are also depicted – climatic data are important in differentiating large scale divisions of ecosystems and ecoregions, which are then further differentiated based on vegetation and terrain.

Total biomass consumed (step 5 in the main text Figure 1 flow chart) was estimated using the assumptions outlined in Spracklen et al., (2009). The fractional fuel consumption as a function of fire severity is provided in Table 4 in Spracklen et al. (2009) (available here: https://agupubs.onlinelibrary.wiley.com/doi/full/10.1029/2008jd010966) and addresses fuel consumption by burn severity for litter and light fuels, medium fuels, heavy fuels, duff, grass, shrub, and canopy. Similar to Yue et al., (2013) we assumed that 25% of the land area burns with high, medium and low intensity and that 25% of the area is left unburned.

Finally, the BC and OC emissions were estimated by applying the biofuel burning emissions factors from Table 1 of Andreae and Merlet (2001) – 0.59 g/kg for BC and 4.0 g/kg for OC.

3. Relative Humidity and Rocky Mountain Ecoregion Regression

A complete estimate of area burned by wildfires requires the input of three additional climate variables: relative humidity (RH), wind speed and air pressure. We expand the LOCA climate dataset to include these variables using a binning approach described in the Technical Appendix to U.S EPA (2017a). Briefly, the binning approach effectively associate historical time series of climate variables to future projections. For example, to project future RH, we associated RH with temperature and precipitation based on historical daily data, additional climate variables, including relative humidity, wind speed and air pressure, using a binning approach. Relative humidity (RH), for example, is required to estimate key inputs for the area burned regressions, in particular the Duff Moisture Code (DMC) and Build-Up Index (BUI) variables, but RH is not available from the LOCA downscaled dataset. The historical pattern of relative humidity cannot be simply repeated to fill in the missing LOCA values, as the arrival times of the LOCA tmax, tmin, and precipitation outputs are drawn from the GCM projections rather than the historical time series. To fill in the missing values, we use a binning approach, which starts with the assumption that there is some relationship between temperature/precipitation and relative humidity. We effectively associate historical daily humidity with daily historical temperature and precipitation, and then used those binned associations to assign projected RH in LOCA future daily time series. Historical humidity for our study area is available from the Princeton Land Surface Hydrology Group (Sheffield et al. 2006).

Yue et al. originally calculated relative humidity (RH) as a function of daily mean temperature and dew point. This approach is different than the one utilized in the LOCA dataset, and resulted in minor differences in these regression variables that were amplified by the area burned equations. To correct for this bias, a set of regression equations were developed to relate each RH predictor variable with temperature and precipitation variables. Then the RH variables were recalculated using those models and the LOCA temperature and precipitation data. While the fire indexes also rely on estimates of daily RH, the effect of these differences was not as significant for those variables, and they were not recalculated. The exception to this was the Rocky Mountain ecoregion, where a threshold effect related to the annual Duff Moisture Code index resulted in consistent underestimation of area burned. As a result, the regression model for that ecoregion was re-derived using the same stepwise regression approach utilized by Yue et al., but using only temperature and precipitation predictor variables.

Additional analysis was performed to improve the performance of the Rocky Mountain ecoregion area burned regressions using LOCA inputs.

a. Linear regression plot (Adj. $R^2 = 0.45$)

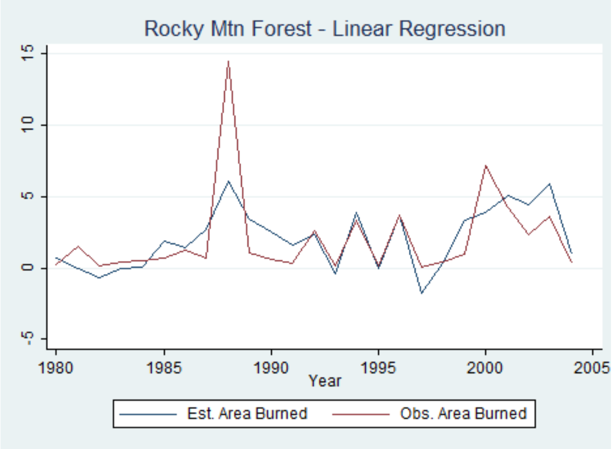

b. Log linear regression plot (Adj. $R^2 = 0.76$)

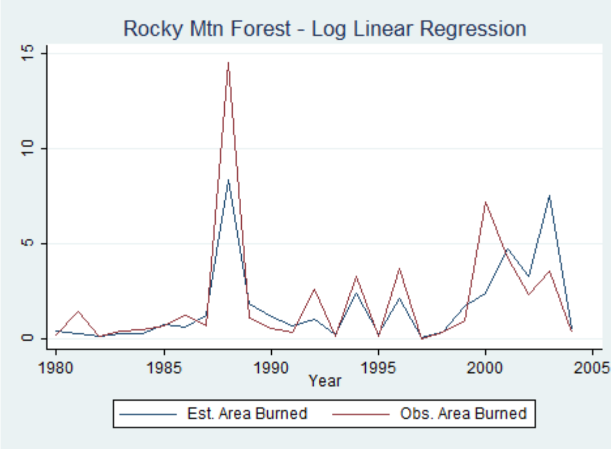

c. Linear regression residuals

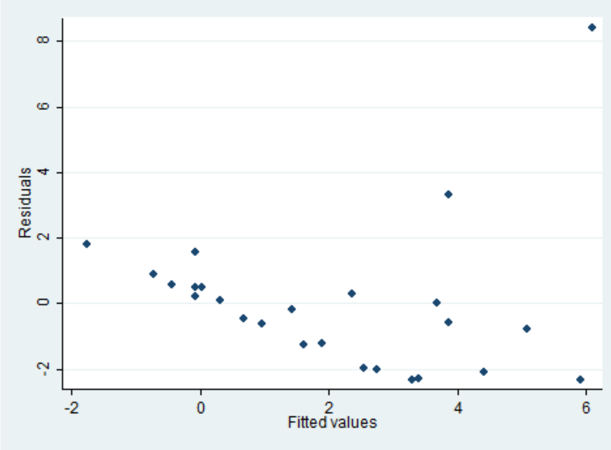

d. Log linear regression residuals

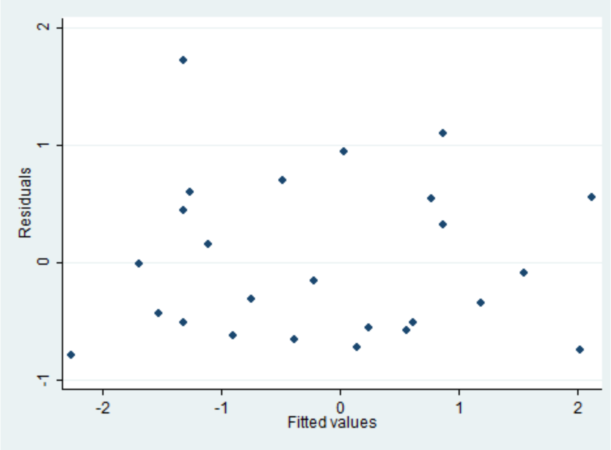

Figure S2. Rocky Mountain Forest regression results for linear and log linear models based on average daily precipitation during the fire season.

a. Linear regression projections

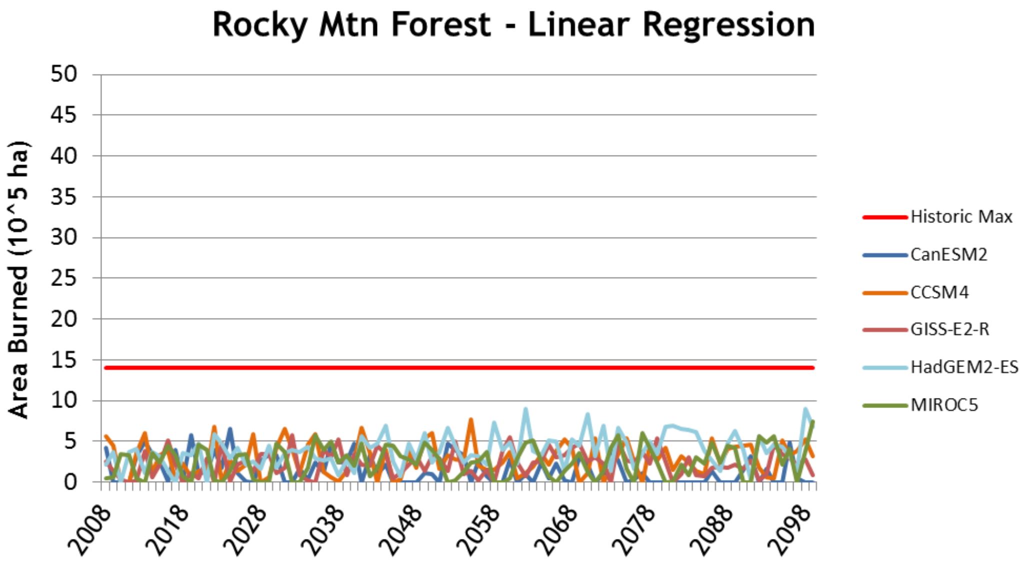

b. Log linear regression projections

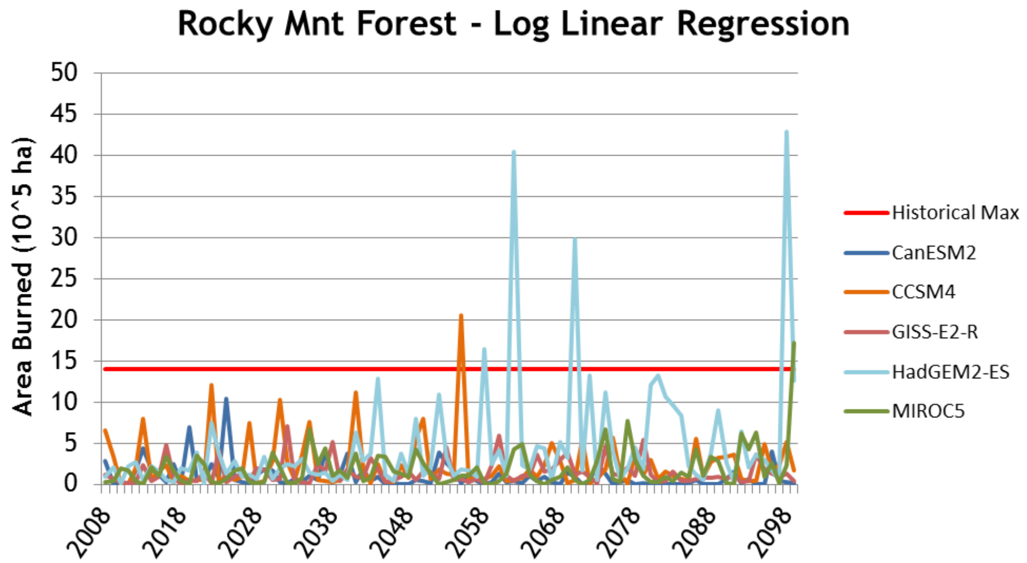

Figure S3. Estimated future area burned for the Rocky Mountain Forest ecoregion calculated using the linear and log linear models with LOCA average daily precipitation during the fire season from each of the five CIRA global climate models.

Table S2. Additional relative humidity regressions

Meteorological Variable	Regression Equation	Adjusted R^2
RH.ANN(-1)	-0.035*BUI(-1) + 0.505*Prec.ANN(-1) + 70.714	0.15
RH.WIN(-1)	0.894*ln(Prec.WIN) + 4.141	0.57
RH.FS(-1)	-0.798*Tmax.SUM(-1) + 10.929*Prec.FS(-1) + 53.342	0.79
RH.ANN	3.86*Prec.SUM + 8.956*Prec.WIN + 0.1*DMCmax + 42.455	0.50

4. LOCA-based Regression Model Performance Against Historic Data

Table S3. Summary of area burned and dry biomass consumption from wildfire - compared to Yue et al. (2013) and historical data

Ecoregion	Annual average area burned (10,000s ha)			Annual mean dry biomass consumption from wildfire (Tg)		
	Observed (1980-2004)	Yue et al. 2013 (1986-2000)	This work (1986-2000)	Observed (1980-2004)	Yue et al. 2013 (1986-2000)	This work (1986-2000)
**Pacific Northwest**	11.2	11.3	10.583	3.94	4.72	2.63
**Nevada Mountains**	28.1	30.1	32.189	1.22	1.72	1.87
**Great Plains**	6.7	6.9	2.457	1.78	0.89	0.28
**California Coastal**	5.4	6.0	5.546	0.64	0.75	0.34
**Desert SW**	7.3	6.5	7.638	0.34	0.25	0.19
**Rocky Mtn Forest**	24.7	35.9	19.347	5.05	8.63	2.74
**TOTAL**	**83.3**	**96.7**	**77.8**	**12.97**	**16.96**	**8.06**

5. Additional Details on Emissions Calculations

To calculate the quantity and spatial distribution of wildfire-related black carbon (BC) and organic carbon (OC) emissions, we followed the process described by Yue et al. (2013). Briefly, we first partitioned annual area burned into monthly totals using the observed historic seasonal pattern within each ecoregion, assuming that this intra-annual temporal pattern will not change significantly over the century. For each month, total area burned was distributed across a 0.5^{o}x 0.5^{o} grid. To reflect observed spatial patterns in area burned, 70% of area burned was randomly distributed to only 10% of the grid cells while maintaining the historic burn fractions across ecosystems from Spracklen et al. (2009). The remaining 30% of area burned was distributed evenly to the remaining 90% of grid cells, also while maintaining historic burn fractions.

We then calculated the total fuel load for each grid cell using the U.S. Forest Service 1 km x1 km fuelbed map used by Yue et al. (2013) (described further in McKenzie et al. 2007 and Ottmar et al. 2007). Similarly, fuel consumption rates for each fuel type were determined using Table 4 in Spracklen et al. (2009) (see: https://agupubs.onlinelibrary.wiley.com/doi/full/10.1029/2008jd010966), and assuming that fires burn with 25% of each high, medium, and low severity, and that 25% of the area is left unburned. Total biomass consumed was then calculated as the product of the total fuel load, the applicable consumption rate, and the area burned for the given grid cell for each month. Finally, BC and OC emissions were calculated by applying the biofuel burning emissions factors published by Andreae and Merlet (2001). Similar to Yue et al. (2013), we did not address

potential effects of wildfires on the biosphere or the impact of climate change on vegetation type and extent.

Table S4: Summary of Wildfire-Attributable Emissions (BC+OC) and Differences from Baseline in the Western US (Gg)

**2000 era (1996-2005)**	81					
	**CanESM2**	**CCSM4**	**GISS-E2-R**	**HadGEM2-ES**	**MIROC5**	**GCM mean**
**RCP 4.5**						
**2050 era (2046-2055)**	92 (+14%)	111 (+37%)	79 (-2%)	111 (+38%)	100 (+25%)	99 (+22%)
**2090 era (2086-2095)**	114 (+41%)	97 (+20%)	80 (-1%)	136 (+68%)	110 (+36%)	107 (+33%)
**RCP 8.5**						
**2050 era (2046-2055)**	105 (+30%)	129 (+60%)	87 (+8%)	138 (+71%)	100 (+23%)	112 (+39%)
**2090 era (2086-2095)**	113 (+40%)	126 (+56%)	95 (+17%)	169 (+109%)	126 (+57%)	126 (+56%)
Note: Values in parentheses are changes relative to the 2000 era total emissions.						

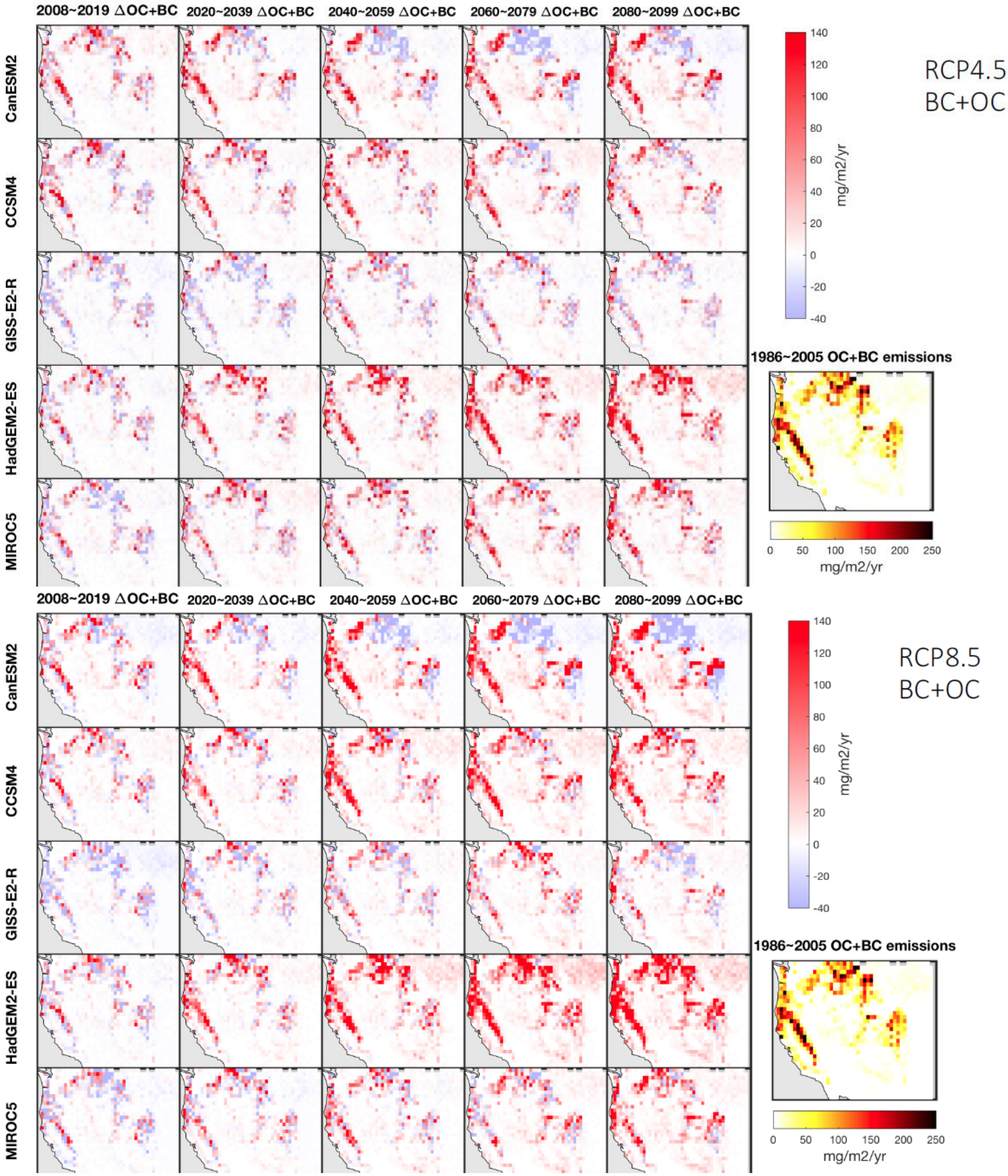

Figure S4: Spatial Summary of BC and OC Emissions Results by GCM and Forecast Era. Top panel is RCP4.5, bottom panel is RCP8.5

6. GEOS-Chem Air Quality Results

Due to the computational expense of conducting 300 model simulation years (30 years, two climate scenarios, and five GCMs), we ran GEOS-Chem with offline chemistry (aerosol-only), which used monthly mean oxidants archived from a previous full-chemistry simulation, including OH, NO3, O3, total nitrate, and production and loss rates for H2O2. The lack of full chemistry feedback in our simulation could affect the concentrations of secondary aerosols (organic and inorganic) that are formed through chemical processes. Therefore, we used secondary aerosol concentrations from a full-chemistry simulation for 2013 conducted by Jun et al. (2019) using the same meteorological data and emissions as this study to correct for the potential bias in the offline simulation. We kept these secondary aerosol concentrations constant to 2013 to isolate the impacts of wildfire BC and OC emissions on PM2.5. Simulated PM2.5 concentrations were highly consistent with observations in both time and space (Figure S5).

Figure S5. Evaluation of the GEOS-Chem simulated $PM_{2.5}$ concentrations with observations. Left: May-Oct monthly medians concentrations from observations and the GEOS-Chem simulation. Observations are averaged over all sites from the Chemical Species Network (CSN) and the IMPROVE network, with the locations outlined on the map (right). The GEOS-Chem simulated concentrations are for the same time period and are collocated with observations. Right: Spatial distribution of $PM_{2.5}$ median concentrations averaged over May-Oct in 1996-2005. Circles represent measurements filled with colors representing observation values. The background is the GEOS-Chem simulated concentration. Statistics are normalized mean bias (NMB) and normalized mean error (NME).

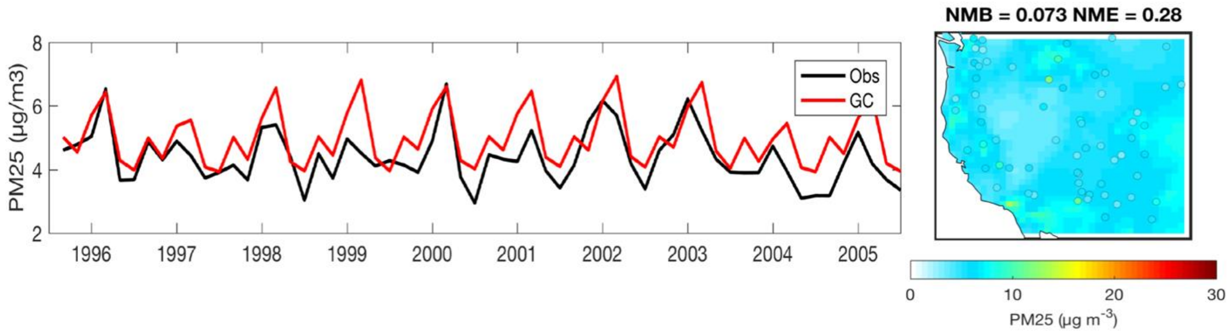

Table S5. Wildfire-Attributable Average PM2.5 Concentrations in the Contiguous US. The estimates are the wildfire-attributable PM2.5 concentrations calculated by subtracting the no-wildfire surface from indicated "with-wildfire" surfaces by GCM and RCP. Spatial average is average of PM2.5 concentrations across all grid cells in contiguous U.S. Population weighted concentrations were weighted by population residing in each grid cell according to year 2000 ICLUS population with a 2010 Census population distribution.

Era	GCM	RCP	Wildfire-Attributable Spatially Weighted Average PM2.5 Conc. in the US (μg/m^3)	Wildfire-Attributable Population Weighted Average PM2.5 Conc. in the US (μg/m^3)
1996-2005	Baseline	Baseline	0.120	0.059
2050 (2046-2055)	CANESM	4.5	0.139	0.077
		8.5	0.161	0.094
	CCSM	4.5	0.169	0.092
		8.5	0.196	0.103
	GISS	4.5	0.118	0.064
		8.5	0.131	0.068
	HADGEM	4.5	0.168	0.088
		8.5	0.208	0.106
	MIROC	4.5	0.152	0.080
		8.5	0.150	0.079
2090 (2086-2095)	CANESM	4.5	0.172	0.101
		8.5	0.170	0.104
	CCSM	4.5	0.144	0.076
		8.5	0.187	0.097
	GISS	4.5	0.120	0.068
		8.5	0.142	0.077
	HADGEM	4.5	0.203	0.107
		8.5	0.254	0.134
	MIROC	4.5	0.165	0.088
		8.5	0.187	0.094

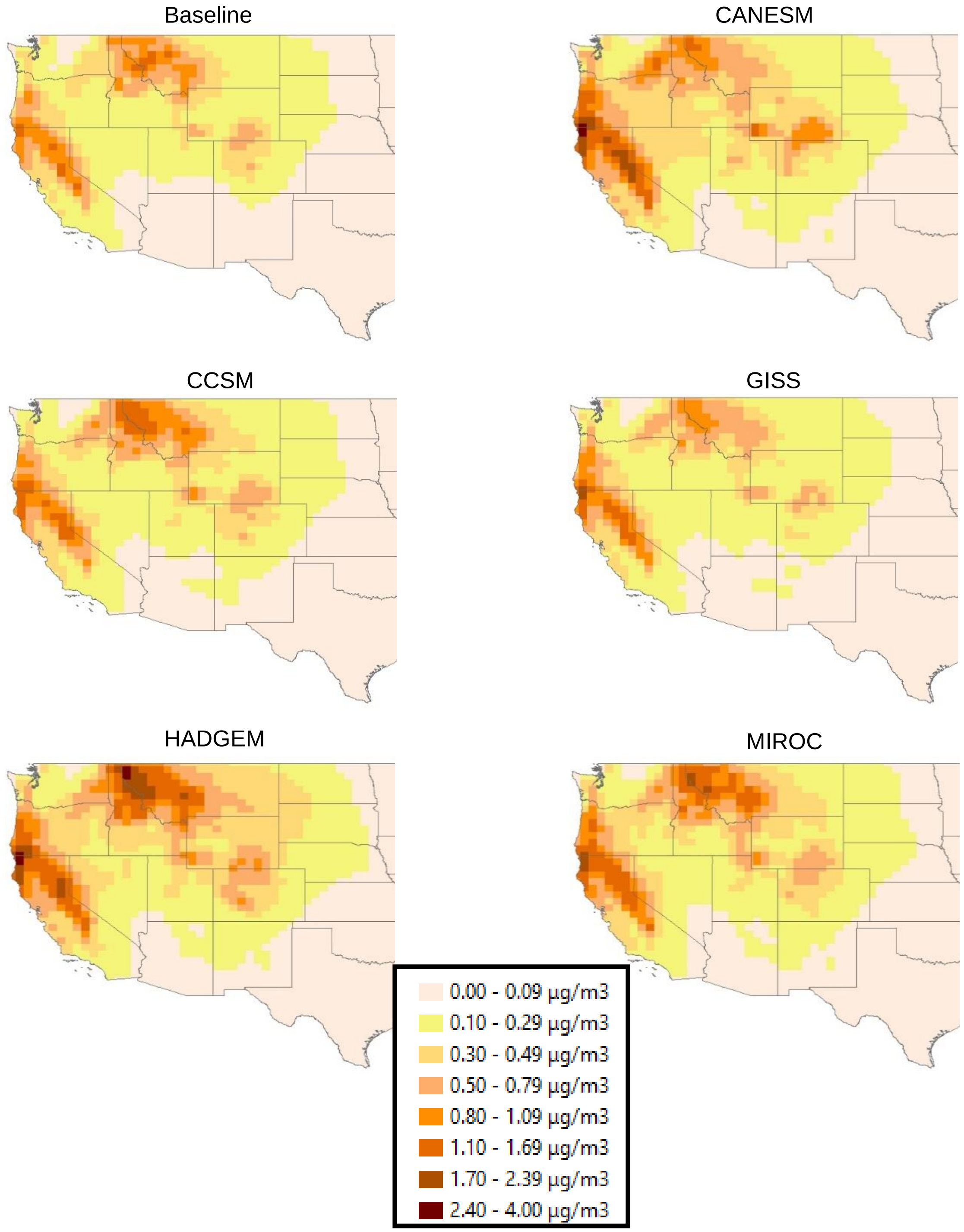

Figure S6. Wildfire-Attributable Annual Average $PM_{2.5}$ Concentrations (µg/m^3) in the Contiguous US for RCP4.5 in the 2090 era. The estimates are the wildfire-attributable $PM_{2.5}$ concentrations calculated by subtracting the no-wildfire surface from indicated "with-wildfire" surfaces by GCM and RCP.

7. BenMAP Modeling and Detailed Health Burden Results

Table S6: Summary of long-term mortality incidence by era, GCM, and RCP, with standard deviation of interannual values within eras

Era	GCM	RCP	Krewski et al. Mean Point Estimate (Ages 30-99)	Krewski et al. Standard Deviation	Lepeule et al. Mean Point Estimate (Ages 25-99)	Lepeule et al. Standard Deviation
1996-2005	Baseline	Baseline	723	40	1639	138
2050 (2046-2055)	CANESM	4.5	1948	105	4378	362
		8.5	2380	128	5347	442
	CCSM	4.5	2319	123	5212	427
		8.5	2620	136	5888	472
	GISS	4.5	1603	88	3605	307
		8.5	1705	89	3836	308
	HADGEM	4.5	2223	122	4997	421
		8.5	2689	141	6040	488
	MIROC	4.5	2019	106	4539	367
		8.5	2000	106	4498	368
2090 (2086-2095)	CANESM	4.5	3073	162	6901	560
		8.5	3147	162	7069	563
	CCSM	4.5	2285	122	5135	422
		8.5	2942	151	6610	524
	GISS	4.5	2056	109	4623	377
		8.5	2339	121	5257	420
	HADGEM	4.5	3217	177	7223	612
		8.5	4071	209	9136	722
	MIROC	4.5	2654	144	5960	497
		8.5	2848	148	6398	512

Table S7: Summary of Health Impact Functions Employed, by Health Endpoint

Health Endpoint	Epidemiological study	Cohort age (years)	Cohort location	Risk Estimate (95% CI)	Function type
Mortality, all-cause	Krewski et al. (2009)	30-99	116 U.S. cities	RR = 1.06 (1.04-1.08) per 10 μg m^{-3}	Log-linear
	Lepeule et al. (2012)	25-99	6 Eastern U.S. cities	RR = 1.14 (1.07-1.22) per 10 μg m^{-3}	Log-linear
	Zanobetti and Schwartz (2009)	0-99	112 U.S. cities	β = 0.000975 (0.000119)	Log-linear
Nonfatal acute myocardial infarction	Peters et al. (2001)	18-99	Boston, MA	OR = 1.62 (1.13-2.34) per 20 μg m^{-3}	Logistic
Hospital admissions, all respiratory (ICD-9 277, 460-465, 466, 480-487, 490, 491, 492, 493, 494, 495, 496, 506, 508, 786)	Delfino et al. (2009)	0-99	Southern California	RR = 1.028 (1.014-1.041) per 10 μg m^{-3}	Log-linear
Hospital admissions, asthma (ICD-9 493)		0-99		RR = 1.048 (1.021-1.076) per 10 μg m^{-3}	Log-linear
Hospital admissions, chronic lung disease (less asthma) (ICD-9 491, 492, 496)		20-99		RR = 1.038 (1.004-1.075) per 10 μg m^{-3}	Log-linear
Emergency hospital admissions, all respiratory (ICD-9 460-519)	Zanobetti et al. (2009)	65-99	26 U.S. communities	β = 0.00207 (0.000446)	Log-linear
Emergency room visits, asthma (ICD-9 493)	Mar et al. (2010)	0-99	Greater Tacoma, WA	RR = 1.04 (1.01-1.07) per 7 μg m^{-3}	Log-linear
	Slaughter et al. (2005)	0-99	Spokane, WA	RR = 1.03 (0.98-1.09) per 10 μg m^{-3}	Log-linear
Acute bronchitis	Dockery et al. (1996)	8-12	24 U.S. and Canadian communities	OR = 1.50 (0.91-2.47) per 14.9 μg m^{-3}	Logistic
Upper respiratory symptoms	Pope et al. (1991)	9-11	Utah Valley	β = 0.0036 (0.0015)	Logistic
Lower respiratory symptoms	Schwartz and Neas (2000)	7-14	6 U.S. cities	OR = 1.33 (1.11-1.58) per 15 μg m^{-3}	Logistic
Asthma exacerbation (cough)	Ostro et al. (2001)	6-18	Los Angeles, CA	OR = 1.03 (0.98-1.07) per 30 μg m^{-3}	Logistic
Asthma exacerbation (wheeze)				OR = 1.06 (1.01-1.11) per 30 μg m^{-3}	Logistic

Health Endpoint	Epidemiological study	Cohort age (years)	Cohort location	Risk Estimate (95% CI)	Function type
Asthma exacerbation (shortness of breath)				OR = 1.08 (1.00-1.17) per 30 μg m^{-3}	Logistic
Asthma exacerbation (cough)	Mar et al. (2004)	6-18	Spokane, WA	OR = 1.21 (1.00-1.47) per 10 μg m^{-3}	Logistic
Asthma exacerbation (shortness of breath)				OR = 1.13 (0.86-1.48) per 10 μg m^{-3}	Logistic
Hospital admissions, all cardiovascular (less myocardial infarction) (ICD-9 390-459)	Zanobetti et al. (2009)	65-99	26 U.S. communities	β = 0.00189 (0.000283)	Log-linear
Hospital admissions, all cardiovascular (less myocardial infarction) (ICD-9 426-427, 428, 430-438, 410-414, 429, 440-449)	Peng et al. (2009)	65-99	119 U.S. counties	β = 0.00068 (0.000214)	Log-linear
Hospital admissions, all cardiovascular (less myocardial infarction) (ICD-9 426-427, 428, 430-438, 410-414, 429, 440-449)	Peng et al. (2008)	65-99	108 U.S. counties	β = 0.00071 (0.00013)	Log-linear
Hospital admissions, all cardiovascular (less myocardial infarction) (ICD-9 426-427, 428, 430-438, 410-414, 429, 440-449)	Bell et al. (2008)	65-99	202 U.S, counties	β = 0.0008 (0.0001)	Log-linear
Hospital admissions, all cardiovascular (less myocardial infarction) (ICD-9 390–429)	Moolgavkar (2000)	20-64	Los Angeles, CA	β = 0.0014 (0.000341)	Log-linear
Work loss days	Ostro (1987)	18-64	Nationwide	β = 0.0046 (0.00036)	Log-linear
Minor restricted activity days (MRADs)	Ostro and Rothschild (1989)	18-64	Nationwide	β = 0.00741 (0.0007)	Log-linear

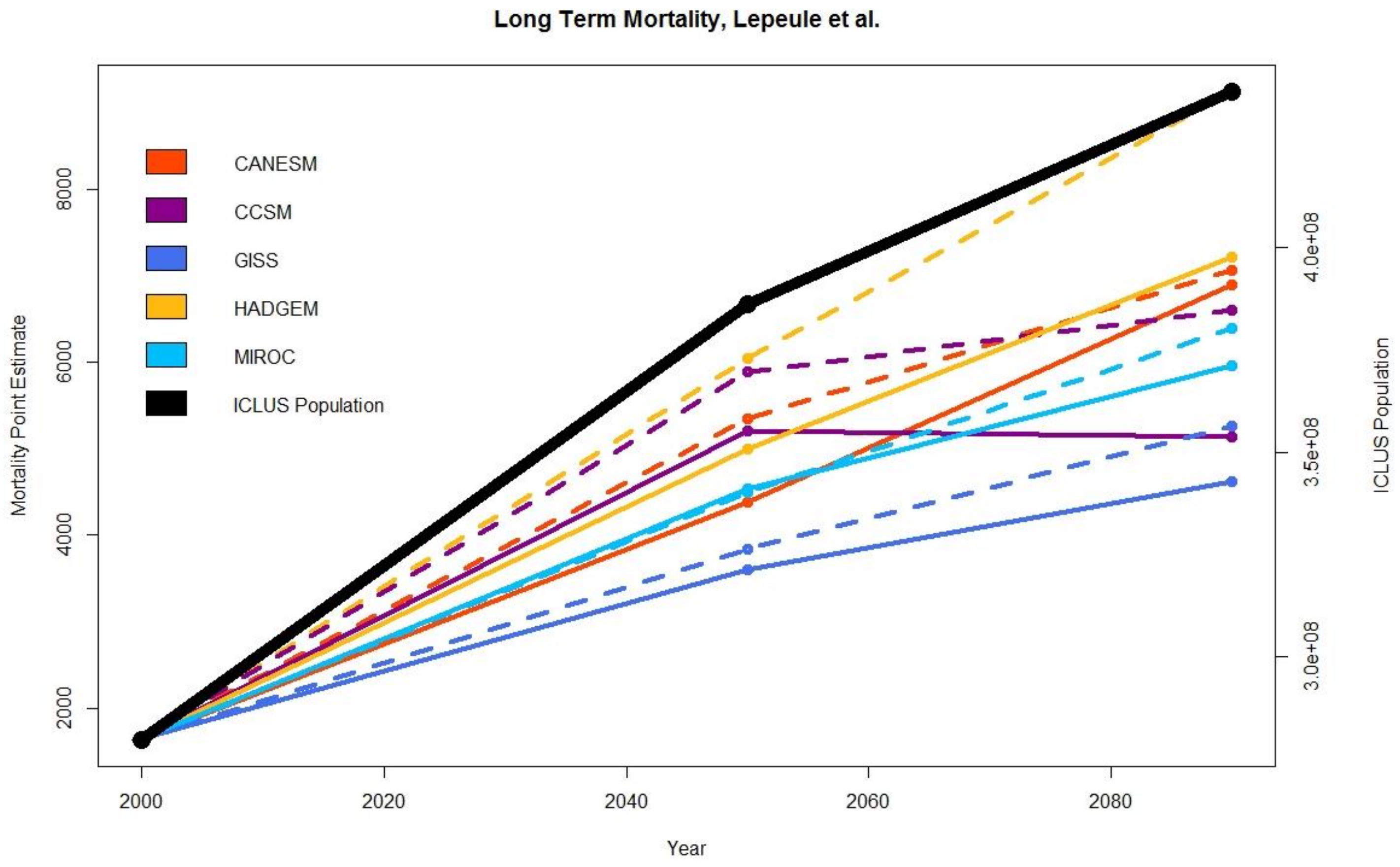

Figure S7. Estimated long-term mortality health burden by GCM compared to trend in population forecast. Thick black line shows ICLUS population estimates, with corresponding axis on the right-hand side. Note that y-axes are not set to zero. Dashed lines are for RCP8.5, solid lines are for RCP4.5. Wildfire risk to mortality grows slower than population from 2000, to 2050, and faster than population from 2050 to 2090 for most GCMs. The exception is CCSM, where increased precipitation in the 2090 period suppresses wildfire risk. Note that mortality impact associated with HADGEM model projection shows continuing increases across the projection period.

The concentration-response relationships used in this analysis are a subset of those used by the EPA in the 2012 $PM_{2.5}$ NAAQS RIA (U.S. EPA 2012). This analysis utilizes ambient $PM_{2.5}$ concentration-response functions for health endpoints with a demonstrated link to wildfire episodes, based on literature review conducted for this research. Other health endpoints without a demonstrated link to wildfire episodes are included as a sensitivity analysis.

Health endpoints in the primary analysis are listed in Tables S9 and S10 and include all-cause mortality, nonfatal acute myocardial infarctions, respiratory hospital admissions, respiratory emergency room visits, and other respiratory effects (acute bronchitis, asthma exacerbation, and upper and lower respiratory symptoms). It should be noted that the all-cause mortality endpoint includes multiple health impact functions that estimate effects from both long-term $PM_{2.5}$

exposure (Krewski et al. (2009); Lepeule et al. (2012)) and short-term $PM_{2.5}$ exposure (Zanobetti and Schwartz (2009)) and that these effects are not additive (for the economic valuation step, only estimates from Krewski et al. are used).

Table S8: Wildfire-Induced Health Burden (Mortality and Morbidity) – Total Incidence Burden (cases per year) for Base Period (1996-2005) and Projected Excess Health Burden Associated with Climate-Induced Changes in Wildfire Activity for 2050 and 2090 Projection Period

Health endpoint	Author	Age (years)	Reference burden (per year)	Future scenario	10-year averaged excess burden relative to reference (per year)	
					2050	2090
Mortality, All Cause (short-term)	Zanobetti and Schwartz	0-99	130	RCP4.5	220 (150,270)	320 (220,420)
				RCP8.5	260 (160,330)	390 (270,560)
Mortality, All Cause (long-term)	Krewski et al.	30-99	720	RCP4.5	1300 (880,1600)	1900 (1300,2500)
				RCP8.5	1600 (980,2000)	2300 (1600,3300)
Mortality, All Cause (long-term)	Lepeule et al.	25-99	1600	RCP4.5	2900 (2000,3600)	4300 (3000,5600)
				RCP8.5	3500 (2200,4400)	5300 (3600,7500)
Acute Myocardial Infarction, Nonfatal	Peters et al.	18-99	500	RCP4.5	1000 (740,1300)	1500 (1100,1900)
				RCP8.5	1200 (820,1500)	1800 (1300,2600)
HA, All Respiratory	Delfino et al.	0-99	350	RCP4.5	640 (430,780)	940 (640,1200)
				RCP8.5	760 (490,960)	1100 (790,1600)
EHA, All Respiratory	Zanobetti et al.	65-99	120	RCP4.5	380 (280,460)	560 (400,700)
				RCP8.5	450 (310,540)	660 (480,910)
HA, Asthma	Delfino et al.	0-99	68	RCP4.5	74 (44,94)	110 (71,150)
				RCP8.5	90 (52,120)	140 (91,200)
HA, Chronic Lung Disease (less Asthma)	Delfino et al.	20-99	69	RCP4.5	150 (100,180)	220 (150,280)
				RCP8.5	180 (120,220)	260 (180,360)
Emergency Department Visits, Asthma	Mar et al., Slaughter et al.	0-99	400	RCP4.5	310 (160,410)	480 (280,670)
				RCP8.5	390 (200,530)	620 (380,940)
Acute Bronchitis	Dockery et al.	8-12	1300	RCP4.5	770 (340,1100)	1300 (680,1800)
				RCP8.5	1000 (440,1400)	1600 (950,2600)

Upper Respiratory Symptoms	Pope et al.	9-11	24000	RCP4.5	14000 (6100,19000)	23000 (12000,33000)
				RCP8.5	19000 (8000,26000)	30000 (17000,47000)
Lower Respiratory Symptoms	Schwartz and Neas	7-14	17000	RCP4.5	9400 (4200,13000)	15000 (8400,22000)
				RCP8.5	12000 (5500,17000)	20000 (12000,31000)
Asthma Exacerbation, Cough	Mar et al., Ostro et al.	6-18	22000	RCP4.5	28000 (5500,62000)	42000 (11000,95000)
				RCP8.5	53000 (7200,100000)	63000 (16000,180000)
Asthma Exacerbation, Shortness of Breath	Mar et al., Ostro et al.	6-18	30000	RCP4.5	17000 (7400,24000)	28000 (15000,41000)
				RCP8.5	23000 (9700,32000)	37000 (21000,58000)
Asthma Exacerbation, Wheeze	Ostro et al.	6-18	46000	RCP4.5	26000 (11000,36000)	43000 (23000,62000)
				RCP8.5	35000 (15000,48000)	56000 (33000,89000)
HA, All Cardiovascular (less Myocardial Infarctions)	Zanobetti et al., Peng et al. (2008), Peng et al. (2009), Bell et al., Moolgavkar	18-99	120	RCP4.5	250 (180,310)	370 (260,480)
				RCP8.5	300 (190,370)	440 (310,620)
Work Loss Days	Ostro	18-64	100000	RCP4.5	57000 (24000,81000)	98000 (52000,140000)
				RCP8.5	77000 (32000,110000)	130000 (74000,200000)
Minor Restricted Activity Days	Ostro and Rothschild	18-64	610000	RCP4.5	340000 (150000,480000)	580000 (320000,830000)
				RCP8.5	460000 (200000,640000)	760000 (440000,1200000)

Table S9: Disaggregation of Mortality, All Cause (long-term) from authors use of Krewski et al. function, into climate-influence and influence of changes in population and baseline mortality rates, relative to baseline estimates.

Era	GCM	RCP	Total mortality point estimate using 2000 Census population (standard deviation)	Influence of climate-attributable wildfires (ratio relative to baseline)	Total mortality point estimate using 2090 ICLUSv2 population (standard deviation)	Influence of population and changes in baseline mortality rates (ratio relative to baseline)
Baseline	Baseline	Baseline	720<br>(40)	--	--	--
2090	CANESM	4.5	1100<br>(59)	1.6	3100<br>(160)	2.7
		8.5	1100<br>(59)	1.6	3100<br>(160)	2.8
	CCSM	4.5	840<br>(45)	1.2	2300<br>(120)	2.7
		8.5	1100<br>(56)	1.5	2900<br>(150)	2.7
	GISS	4.5	750<br>(40)	1.0	2100<br>(110)	2.7
		8.5	860<br>(45)	1.2	2300<br>(120)	2.7
	HADGEM	4.5	1200<br>(66)	1.7	3200<br>(180)	2.7
		8.5	1500<br>(78)	2.1	4100<br>(210)	2.7
	MIROC	4.5	990<br>(54)	1.4	2700<br>(140)	2.7
		8.5	1100<br>(55)	1.5	2800<br>(150)	2.7

For mortality endpoints, the EPA's Guidelines for Preparing Economic Analyses recommends a VSL of $7.9 million ($2008) based on 1990 incomes (U.S. EPA, 2014). To create a VSL using $2015 and based on 2010 incomes, the standard value was adjusted for inflation and income growth based on the approach described in EPA's BenMAP-CE model and its documentation (U.S. EPA, 2018a,b). The resulting value, $9.7 million for 2010 (2015$), was adjusted to future years by assuming an income elasticity of VSL of 0.4 (since personal income is not projected, GDP per capital is used as a proxy for mean personal income). Projections of U.S. population change are described in the main text, while the Emissions Predictions and Policy Analysis model (version 6; Chen et al., 2015) was run to generate a projection of GDP growth. Applying this standard approach yields the following VSL values: $12.4 million in 2050, and $15.2 million in 2090 (in undiscounted 2015$). As more recent literature favors a higher estimate of income

elasticity of VSL, we also perform a sensitivity test using an elasticity of 1.0, reflecting proportional growth in VSL with gross domestic product per capita. Using an elasticity of 1.0 compared with 0.4, our estimated costs associated with the mortality burdens are 87% higher in 2090. The sensitivity analysis suggests that our valuation of the mortality endpoints may be conservative.

All morbidity endpoint estimates were adjusted from 2007$ to 2015$ using BenMAP-CE's default inflation index. Note that the morbidity valuation performed in this analysis is consistent with the valuation methodology used by the EPA in the 2012 $PM_{2.5}$ NAAQS RIA (U.S. EPA 2012). Full valuation results are presented in Table S10.

Table S10. Detailed Health Effect Valuation Results (millions 2005$). Average across five GCMs is presented, with the range across GCMs provided in parentheses below.

Health endpoint	Health Effect Study Author	Age (years)	Reference value (per year)	Future scenario	10-year averaged excess damage relative to reference (per year)	
					2050	2090
Mortality, All Cause (short-term)	Zanobetti and Schwartz	0-99	1200	RCP4.5	3100 (2200,3700)	5600 (4100,7100)
				RCP8.5	3600 (2400,4500)	6700 (4800,9300)
Mortality, All Cause (long-term)	Krewski et al.	30-99	6900	RCP4.5	18000 (13000,22000)	34000 (24000,42000)
				RCP8.5	22000 (14000,27000)	40000 (29000,55000)
Mortality, All Cause (long-term)	Lepeule et al.	25-99	16000	RCP4.5	41000 (29000,49000)	75000 (55000,94000)
				RCP8.5	48000 (32000,60000)	89000 (64000,120000)
Acute Myocardial Infarction, Nonfatal (3% Discount Rate)	Peters et al.	18-99	45	RCP4.5	160 (120,190)	220 (160,270)
				RCP8.5	180 (130,220)	260 (190,350)
Acute Myocardial Infarction, Nonfatal (7% Discount Rate)	Peters et al.	18-99	43	RCP4.5	150 (110,180)	210 (160,270)
				RCP8.5	180 (130,210)	250 (190,340)
HA, All Respiratory	Delfino et al.	0-99	7.4	RCP4.5	26 (19,31)	36 (26,46)
				RCP8.5	30 (21,36)	42 (31,58)
EHA, All Respiratory	Zanobetti et al.	65-99	2.7	RCP4.5	16 (12,18)	22 (16,27)
				RCP8.5	18 (13,21)	26 (19,35)
HA, Asthma	Delfino et al.	0-99	0.81	RCP4.5	1.9 (1.3,2.3)	2.6 (1.8,3.4)
				RCP8.5	2.2 (1.5,2.7)	3.1 (2.2,4.4)
HA, Chronic Lung Disease (less Asthma)	Delfino et al.	20-99	1.1	RCP4.5	4.5 (3.3,5.3)	6.2 (4.5,7.8)
				RCP8.5	5.2 (3.6,6.3)	7.3 (5.3,10)

Health endpoint	Health Effect Study Author	Age (years)	Reference value (per year)	Future scenario	10-year averaged excess damage relative to reference (per year)	
					**2050**	**2090**
Emergency Department Visits, Asthma	Mar et al., Slaughter et al.	0-99	0.12	RCP4.5	0.22 (0.15,0.28)	0.31 (0.21,0.4)
				RCP8.5	0.27 (0.17,0.33)	0.38 (0.26,0.54)
Acute Bronchitis	Dockery et al.	8-12	0.49	RCP4.5	0.54 (0.33,0.68)	0.78 (0.49,1)
				RCP8.5	0.66 (0.38,0.85)	0.96 (0.63,1.4)
Upper Respiratory Symptoms	Pope et al.	9-11	0.62	RCP4.5	0.68 (0.41,0.86)	0.98 (0.62,1.3)
				RCP8.5	0.83 (0.47,1.1)	1.2 (0.79,1.8)
Lower Respiratory Symptoms	Schwartz and Neas	7-14	0.27	RCP4.5	0.29 (0.18,0.37)	0.42 (0.27,0.56)
				RCP8.5	0.35 (0.21,0.46)	0.52 (0.34,0.76)
Asthma Exacerbation, Cough	Mar et al., Ostro et al.	6-18	0.99	RCP4.5	2 (0.65,4)	2.8 (0.99,5.9)
				RCP8.5	3.4 (0.75,6.2)	4.1 (1.3,11)
Asthma Exacerbation, Shortness of Breath	Mar et al., Ostro et al.	6-18	1.3	RCP4.5	1.4 (0.87,1.8)	2.1 (1.3,2.8)
				RCP8.5	1.8 (1,2.3)	2.6 (1.7,3.9)
Asthma Exacerbation, Wheeze	Ostro et al.	6-18	2	RCP4.5	2.2 (1.3,1.8)	3.2 (2,4.3)
				RCP8.5	2.7 (1.5,3.5)	4 (2.6,5.9)
HA, All Cardiovascular (less Myocardial Infarctions)	Zanobetti et al., Peng et al. (2008), Peng et al. (2009), Bell et al., Moolgavkar	18-99	3.3	RCP4.5	13 (9.8,16)	19 (14,23)
				RCP8.5	15 (11,18)	22 (16,30)
Work Loss Days	Ostro	18-64	13	RCP4.5	15 (9.2,19)	22 (14,29)
				RCP8.5	18 (11,24)	27 (18,40)
Minor Restricted Activity Days	Ostro and Rothschild	18-64	32	RCP4.5	34 (21,44)	51 (33,69)
				RCP8.5	42 (24,55)	64 (41,94)

Presented in Millions USD (2015$).

8. Wildfire Response Costs

In addition to estimating the health impacts of wildfires through contributions to particulate matter air pollution, we also estimated changes in wildfire response costs. The method used is consistent with that described in Mills et al. (2014), applying data on the average, regional response cost estimates from the National Wildfire Coordinating Group (NWCG), available at the web portal for National Geographic Area Coordination Center – National Interagency Fire Center (GACC-NIFC). NWCG compiles information in ICS-209 reports for "Significant incidents," which reflect

wildfires burning 100+ acres or involving Incident Management Teams of type 1 or 2. We used geographic coordinate information in the web portal database to compile response costs at the state level, and assigned average response costs by state to our area burned estimates described above to generate total wildfire response costs for our projection period, as well as annual estimates, for each of the five GCMs and two RCPs. Response costs used in Mills et al. (2014) reflected data available for 2002 – 2012; we updated the average regional response costs using newly available data for 2002 – 2018, which led to a slight increase in the average response costs for most regions, and larger increases for the California regions.

This analysis used data from the National Wildfire Coordinating Group (NWCG) to monetize the projected changes in acreage burned by wildfires. Specifically, the analysis developed spatially resolved wildfire response costs from NWCG data on the size (i.e., acres burned), origin, and total response costs for distinct wildfires in the contiguous United States (U.S.) from 2002 to 2018. Figure S8 provides the Geographic Area Coordination Center (GACC) boundaries used by the NWCG to coordinate wildfire responses and to collect and report wildfire-related data (GACC 2011).

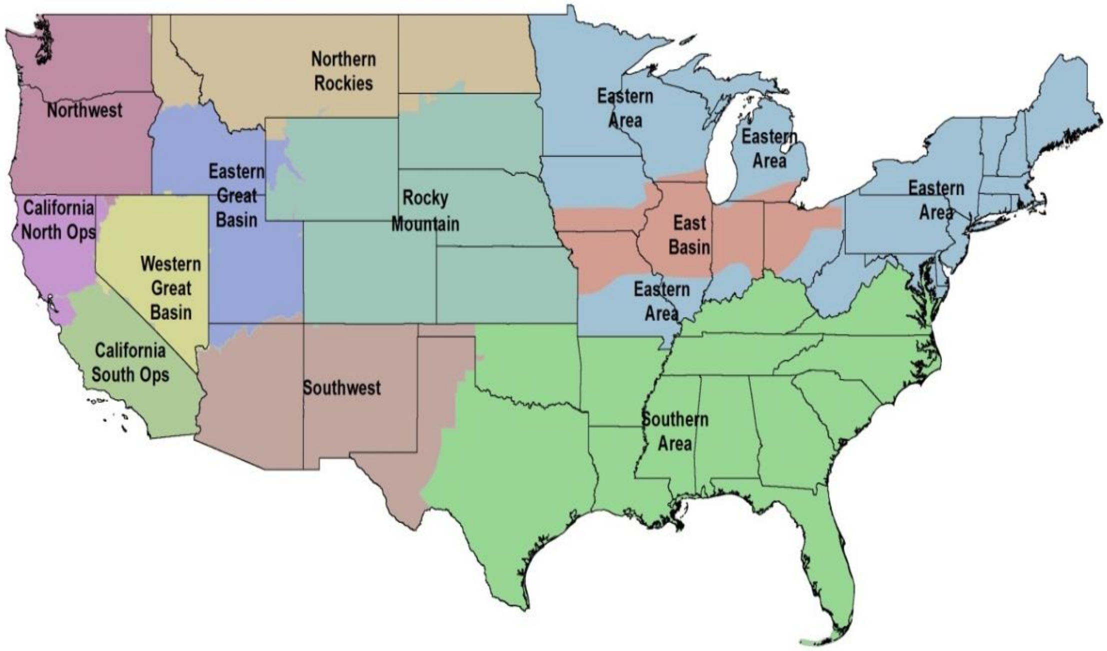

Figure S8. GACC fire region boundaries

The NWCG provides ICS-209 report summaries for 2002-2018, for each GACC region. Mills et al. 2014 also used this data source, but with an earlier vintage which provided costs for the 2002 – 2010 period.

Duplication Adjustments: Duplicate fires, with the same incident number, incident name, and start date were adjusted. The cost value that was the larger of the two was assumed to be cumulative and used for our calculation purposes. A total of 191 duplicates existed, of which only 23 had differing cost values.

Great Basin region: In the years 2015-2018, Western Great Basin and Eastern Great Basin were combined. We manually altered the coding to reflect the two, WB and EB. According to the GACC, Nevada made up the Western Great Basin whereas the other states of the Great Basin region were part of the Eastern Great Basin.

Wildfire Mapping: Wildfires are mapped in ArcGIS based on latitude and longitude information provided in ICS-209 forms. Less than one percent of all fires do not have latitude and longitude. Those fires are excluded from the analysis. Fires with locations that do not fall within the contiguous US were also excluded. Some of these fires likely had latitudes and longitudes with errors (such as those placed within the Arctic Circle), but there was no clear pattern to the errors and thus the location data for these fires could not be reliably corrected. After excluding fires outside the contiguous US or without spatial information, 20,812 fires remain in the analysis. Each of these fires is assigned to a LOCA 0.5^o x 0.5^o grid cell.

Table S11. Results for Wildfire Response Analysis, Cumulative and Annual Estimates

National Climate Assessment Region	Cumulative Acres Burned (millions of acres)		Cumulative Response Costs (millions of $2015, discounted at 3%)	
	RCP8.5	RCP4.5	RCP8.5	RCP4.5
Northern Plains	49	45	$4,500	$4,300
Southern Plains	3	3	$14	$14
Southwest	150	130	$20,000	$18,000
Northwest	80	73	$6,500	$6,100
**Total**	**280**	**250**	**$31,000**	**$29,000**
Period			Annual Response Costs (millions of $2015, undiscounted)	
**2050 (avg over 2046-2055)**			**$730**	**$640**
**2090 (avg. over 2086-2095)**			**$890**	**$710**

Note: Estimates are averages over results for each of the full suite of GCMs applied, rounded to two significant figures.
